# Distinct genomic landscape of Chinese pediatric acute myeloid leukemia impacts clinical risk classification

**DOI:** 10.1038/s41467-022-29336-y

**Published:** 2022-03-28

**Authors:** Ting Liu, Jianan Rao, Wenting Hu, Bowen Cui, Jiaoyang Cai, Yuhan Liu, Huiying Sun, Xiaoxiao Chen, Yanjing Tang, Jing Chen, Xiang Wang, Han Wang, Wubin Qian, Binchen Mao, Sheng Guo, Ronghua Wang, Yu Liu, Shuhong Shen

**Affiliations:** 1 Key Laboratory of Pediatric Hematology & Oncology of the Ministry of Health of China, Department of Hematology & Oncology, Shanghai Children’s Medical Center, School of Medicine, Shanghai Jiao Tong University, Shanghai, China; 2 Pediatric Translational Medicine Institute, Shanghai Children’s Medical Center, School of Medicine, Shanghai Jiao Tong University, Shanghai, China; 3 Crown Bioscience Inc., Suzhou, Jiangsu China

**Keywords:** Cancer genomics, Acute myeloid leukaemia, Acute lymphocytic leukaemia, Paediatric cancer

## Abstract

Studies have revealed key genomic aberrations in pediatric acute myeloid leukemia (AML) based on Western populations. It is unknown to what extent the current genomic findings represent populations with different ethnic backgrounds. Here we present the genomic landscape of driver alterations of Chinese pediatric AML and discover previously undescribed genomic aberrations, including the *XPO1*-*TNRC18* fusion. Comprehensively comparing between the Chinese and Western AML cohorts reveal a substantially distinct genomic alteration profile. For example, Chinese AML patients more commonly exhibit mutations in *KIT* and *CSF3R*, and less frequently mutated of genes in the RAS signaling pathway. These differences in mutation frequencies lead to the detection of previously uncharacterized co-occurring mutation pairs. Importantly, the distinct driver profile is clinical relevant. We propose a refined prognosis risk classification model which better reflected the adverse event risk for Chinese AML patients. These results emphasize the importance of genetic background in precision medicine.

## Abstract (web summary)

The genomic landscape of pediatric acute myeloid leukemia (AML) has mostly been characterised for Western populations. Here, the authors identify potential driver alterations in Chinese pediatric AML, which differ from Western populations, and propose a prognostic risk classification model.

## Introduction

Among childhood cancers, leukemia is the most common malignancy^1^, and 15–20% of childhood leukemias are acute myeloid leukemia (AML). Although less common than acute lymphoblastic leukemia (ALL), patients with AML have inferior outcomes^2,3^. Genomic profiling based on next-generation sequencing (NGS) has elucidated the genomic landscape of pediatric AML and detected key molecular alterations^4–8^. Such alterations include fusions, like *RUNX1*-*RUNX1T1* and *CBFB*-*MYH11*; *KMT2A* rearrangements; *NUP98* rearrangements; and sequence mutations, like *FLT3*, *KIT*, *WT1*, *CEBPA*, and *NPM1*, which are among the most commonly mutated genes in pediatric AML. Further studies have been conducted to establish the association between key driver alterations and patient outcome. For example, *KMT2A* rearrangements or *FLT3* ITD variants are associated with adverse outcome^9–11^, while *RUNX1*-*RUNX1T1* fusion or mutations in *NPM1* likely indicate a favorable prognosis^12,13^. These findings have substantially improved our understanding of the genetics and molecular complexity underlying AML and have also promoted the development of clinical methods for precision diagnosis and patient management.

Recent studies have revealed that genomic profiles significantly differ between pediatric and adult cancer, including AML^4,5,14–16^. Compared to adult, pediatric AML tends to exhibit higher mutation frequencies in *MYC* ITD and *WT1*, and less frequent mutations in *DNMT3A* and *TP53*. Meanwhile, studies in adult solid tumors have also shown that different ethnic backgrounds may have a profound impact on molecular drivers of disease development and progression^17,18^. Following this evidence, it is notable that although comprehensive genomic studies have revealed key genomic aberrations in pediatric AML, these observations have been primarily based on genomic profiling of patients from Western populations. There remains a lack of genomic profiling in Chinese AML patients, such that precision medicine in Chinese AML is largely biased.

In the present study, we report the comprehensive genomic and transcriptomic study on Chinese pediatric AML. We perform transcriptome sequencing to analyze the driver alterations in 292 pediatric AML cases and their correlations. We further identify a distinct driver profile by comparing our results to the mutation profile characterized in the Children’s Oncology Group-National Cancer Institute TARGET AML initiative, representing the Western pediatric AML cohort^4^. Finally, we demonstrate that the different driver profile identified in Chinese AML is clinic relevant, and we propose a refined risk classification model based on these results.

## Results

### Clinical characteristics of Chinese pediatric AML

We analyzed genomic alterations by studying 292 Chinese pediatric AML patients who were diagnosed and treated at Shanghai Children’s Medical Center (SCMC) from 2001–2018, and for whom adequate material from tumor cells was available (Supplementary Data 1). The clinical characteristics of the Chinese cohort were comparable to those of Western populations^4^. However, Chinese AML patients were younger, with a median age of 5.3 years compared to 10.6 years in the Western cohort (Supplementary Data 2). Patients enrolled in this study were treated on AML-SCMC-2009-A protocol (*n* = 196, 67.1%), AML-SCMC-2009-B (*n* = 78, 26.7%), SCMC-AML-XH99 (*n* = 10, 3.4%), and other (*n* = 6, 2.1%), while information was missing for two patients (0.7%). No significant difference in patient outcome was observed between AML-SCMC-2009-A and AML-SCMC-2009-B (*P* = 0.207) or between patients treated over period of time (2009–2013 versus 2014-2018, *P* = 0.655) (Methods section and Supplementary Fig. 1). Transcriptome sequencing (RNA-seq) was applied to all tumor samples (Supplementary Data 3) and analyzed for both sequence mutations and gene rearrangements (Methods section).

### Chimeric fusions identified in Chinese pediatric AML

**Fig. 1:**
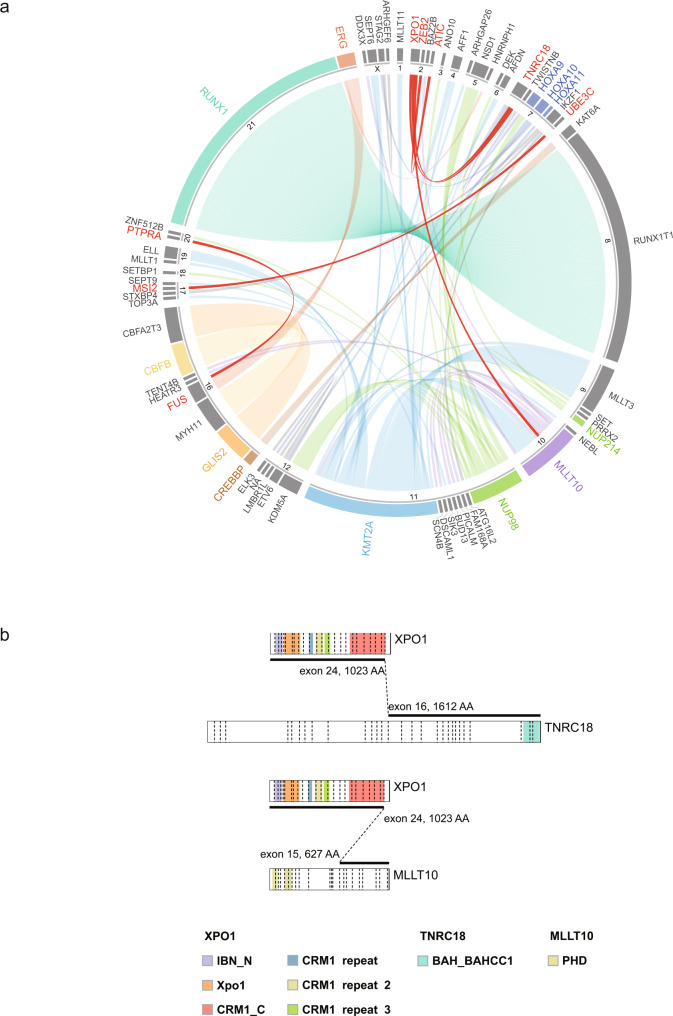
Gene fusions in Chinese pediatric AML. **a** Circos plot shows the previously undescribed and recurrent fusions (*n* = 206), for which one or both fusion partner genes were detected in ≥2 cases. Fusions with multiple partners are grouped and color coded. Fusions that have not been described previously are shown in red. **b** *XPO1* fusions that were detected with RNA-seq, including the in-frame fusion of *XPO1-TNRC18* and *XPO1-MLLT10*.

Analysis of RNA-seq data revealed 224 rearrangements involving 97 genes, in 200 out of 292 patients (68.5%) (Fig. 1a and Supplementary Data 4). In concordance with previous reports, the most prevalent fusions detected in Chinese AML patients included *RUNX1-RUNX1T1* (*n* = 82, 28.1%), *KMT2A* rearrangements (*n* = 45, 15.4%), and *NUP98* rearrangements (*n* = 17, 5.8%). Additionally, we identified a recurrent in-frame fusion involving the *XPO1* gene in three patients in our cohort (Fig. 1b), including the *XPO1-TNRC18* fusion (*n* = 2) and *XPO1-MLLT10* fusion (*n* = 1). Notably, the *XPO1-TNRC18* fusion was also detected in an additional two AML patients who were recently diagnosed at SCMC. Interestingly, all four cases carrying *XPO1*-*TNRC18* belonged to the M7 group and did not harbor any known AML-driving fusion, supporting that this fusion was the potential driver of AML in these cases, and might define a previously unclassified molecular subtype within this FAB group. The *XPO1* gene is mainly involved in the nuclear export of proteins and RNAs. Hotspot mutations in *XPO1* have been described in several tumor types, including B-cell malignancies, and are reportedly associated with tumorigenesis^19–22^. On the other hand, the functions of *TNRC18* involve chromatin and DNA binding, and have not been associated with tumors. The *XPO1*-*TNRC18* chimeric fusion protein conserved both the exportin 1-like domain of XPO1 and the bromo-adjacent homology domain of TNRC18. Further experimental investigation is required to elucidate the function of this fusion protein. Overall, the cases with *XPO1*-*TNRC18* accounted for 5.0% of the AML M7 subtype (2 of 40 in our cohort). We also identified other gene rearrangements in our current analysis that have not been observed previously, including *PTPRA-FUS, ZEB2-ATIC*, and *MSI2-UBE3C* (*n* = 1 each).

### Distinct profile of sequence mutations in Chinese pediatric AML

We optimized the variant calling and processing pipeline for analyzing potential somatic and cancer-associated sequence mutations from tumor-only RNA-seq data (Methods section). To first evaluate the performance of this approach, we applied it to RNA-seq data collected from 10 previously published pediatric ALL cases^23^, for which we also had matched whole-genome sequencing (WGS) data for both tumor and remission samples from each case. Results demonstrated that our analysis of tumor-only RNA-seq data successfully identified 85.7% of the driver mutations discovered by WGS (18 of 21) (Supplementary Data 5 and Supplementary Fig. 2a–d). Meanwhile, RNA-seq analysis detected an additional nine potential driver mutations, including NRAS G13D and KRAS G13D, among others. Of these 9 mutations, 8 had been included in the capture validation experiment using genomic DNA in our previous study. Among these 8 mutations, 7 (87.5%) (Supplementary Data 5 and Supplementary Fig. 2e) were successfully validated. Notably, all nine mutations were subclonal (vaf < 0.3) and were missed in WGS analysis due to insufficient coverage. These results demonstrate the power of analyzing sequence mutations from RNA-seq, especially for detection of subclonal variants.

**Fig. 2:**
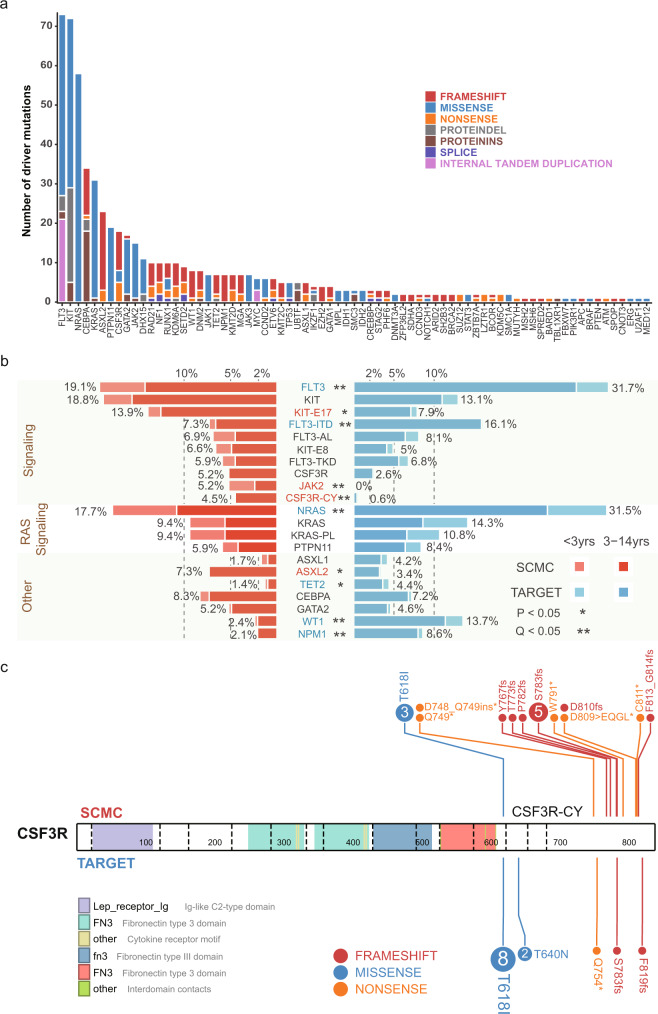
Genes targeted by driver sequence mutation in Chinese pediatric AML. **a** Bar-plot shows driver mutations ranked in order of recurrence. Colors represent different mutation types. **b** Differing driver mutation recurrence between East and West pediatric AML cohorts. Only patients of <15 years old were included, for consistency between the Chinese (*n* = 288 cases) and Western (*n* = 498 cases) cohorts. For mutation hot-spots, we included only cases analyzed with high-throughput sequencing (*n* = 288 for Chinese cohort; *n* = 482 for Western cohort). Butterfly plot shows genes with mutation recurrence >4% in the SCMC or TARGET cohort. Genes are grouped into pathways and ranked in order of recurrence within each pathway. Left (red), Mutation recurrences in the SCMC cohort. Right (blue), Mutation recurrences in the TARGET cohort. Patients were breakdown by <3 years (light color) and 3–14 years (dark color) age group in each cohort. Asterisk indicates the statistical significance (*P* < 0.05, two-sided Fisher’s exact test, Q: FDR-adjusted *P*. Exact *P* and *Q* values were present in Supplementary Data 8a.) for each gene. Genes showing higher mutation recurrence in the SCMC or TARGET cohort are labeled in red or blue, respectively; black indicates no significant difference. Recurrence is labeled besides each bar. KIT-E17: *KIT* exon 17; KIT-E8: *KIT* exon 8; FLT3-ITD: *FLT3* internal tandem duplication; FLT3-TKD: tyrosine kinase domain (amino acids 835 and 836) of FLT3; FLT3-AL: active loop (amino acids 829–858) of FLT3; KRAS-PL: P-loop (amino acids 10–17) of KRAS; CSF3R-CY: cytoplasmic (amino acids 651–831) of CSF3R. **c** Driver mutations targeting different hot-spots in Chinese and Western pediatric AML. Compared to the Western cohort (TARGET), Chinese AML cases (SCMC) showed a higher mutation frequency in the CSF3R cytoplasmic domain (amino acids 651–831). Mutations are shown along the gene, with the amino acid change labeled. The number inside each circle indicates the number of cases carrying the mutation, while colors represent mutation types.

Using this method, we next identified a total of 975 nonsynonymous sequence mutations affecting 305 genes (Supplementary Data 6). These mutations included 707 single-nucleotide variants (SNV) and 268 insertion/deletion (indels), with a median of four mutations detected per case (range 0–10). We further analyzed potential driver mutations by applying a combined strategy, integrating mutation pathogenicity analysis using PeCanPIE^24^ with mutation cluster analysis using MutClan (Methods section and Supplementary Data 6). A total of 572 potential driver variations were identified affecting 73 genes. Moreover, 24 internal tandem duplications (ITD, Supplementary Data 7) were detected with CICERO^25^, influencing *FLT3* and *MYC*. Overall, driver sequence mutations were detected in 81.8% of cases (*n* = 239), with a median of three driver mutations detected per case (range, 0–8). Our subsequent analysis focused only on driver mutations. Among the drivers, 10 genes were recurrently mutated in over 5% of patients (Fig. 2a), including *FLT3* (*n* = 56 patients, 19.2%), *KIT* (*n* = 54, 18.5%), *NRAS* (*n* = 52, 17.8%), *KRAS* (*n* = 27, 9.2%), *CEBPA* (*n* = 24, 8.2%), *ASXL2* (*n* = 21, 7.2%), *PTPN11* (*n* = 18, 6.2%), *CSF3R* (*n* = 15, 5.1%), *GATA2* (*n* = 15, 5.1%), and *JAK2* (*n* = 15, 5.1%). Notably, we uncovered driver genes in pediatric AML, including mutations in *LZTR1* (*n* = 2) and *SPOP* (*n* = 1), which have not been previously associated with pediatric AML, as well as loss of function mutations in *ARID2* (*n* = 2) and *SH2B3* (*n* = 2), which have been reported as pathogenic in other pediatric cancers, like ALL, but not in AML.

Although genomic mutations in most of these genes have been previously reported in pediatric AML, we observed a dramatically different profile of mutation occurrence in Chinese cohort compared to in the TARGET AML study representing the Western population. Among the 21 genes or hotspots within a driver gene recurrently mutated in >4% of patients in either SCMC or TARGET cohort, 10 (47.6%) showed a significantly different mutation frequency (*P* < 0.05, two-sided Fisher’s exact test) (Fig. 2b, c and Supplementary Fig. 3 and Supplementary Data 8a). Among these, four showed a higher mutation frequency in Chinese patients, including *ASXL2* (7.3% vs. 3.4% in the Chinese and Western cohorts, respectively, *P* = 0.025), *JAK2* (5.2% vs. 0.0%, *P* < 0.001), *CSF3R* cytoplasmic domain (*CSF3R-CY*, 4.5% vs. 0.6%, *P* < 0.001), and *KIT* exon 17 (*KIT-E17*, 13.9% vs. 7.9%, *P* = 0.020). On the other hand, the Chinese patients showed less frequent mutations in *FLT3* (19.1% vs. 31.7%, *P* = 0.003) and *FLT3* ITD (7.3% vs. 16.1%, *P* = 0.002), *NRAS* (17.7 vs. 31.5%, *P* < 0.001), *WT1* (2.4% vs. 13.7%, *P* < 0.001), *NPM1* (2.1% vs. 8.6%, *P* < 0.001), and *TET2* (1.4% vs. 4.4%, *P* = 0.036). Notably, the different mutation frequency between Chinese and Western patients were mostly contributed by patients of 3-14 years old (Supplementary Fig. 4a and Supplementary Data 8a). Furthermore, we found that *FLT3* and *NRAS* mutations were more frequently detected in younger patients in Chinese cohort (*FLT3*, 14 out of 55, 25.5%; *NRAS*, 20 out of 51, 39.2%) than in TARGET (*FLT3*, 20 out of 158, 12.7%, *P* = 0.033; *NRAS*, 37 out of 157, 23.6%, *P* = 0.046, Supplementary Fig. 4b and Supplementary Data 8b).

### Landscape of driver genomic alterations unveiled different pattern of co-mutations in Chinese pediatric AML

**Fig. 3:**
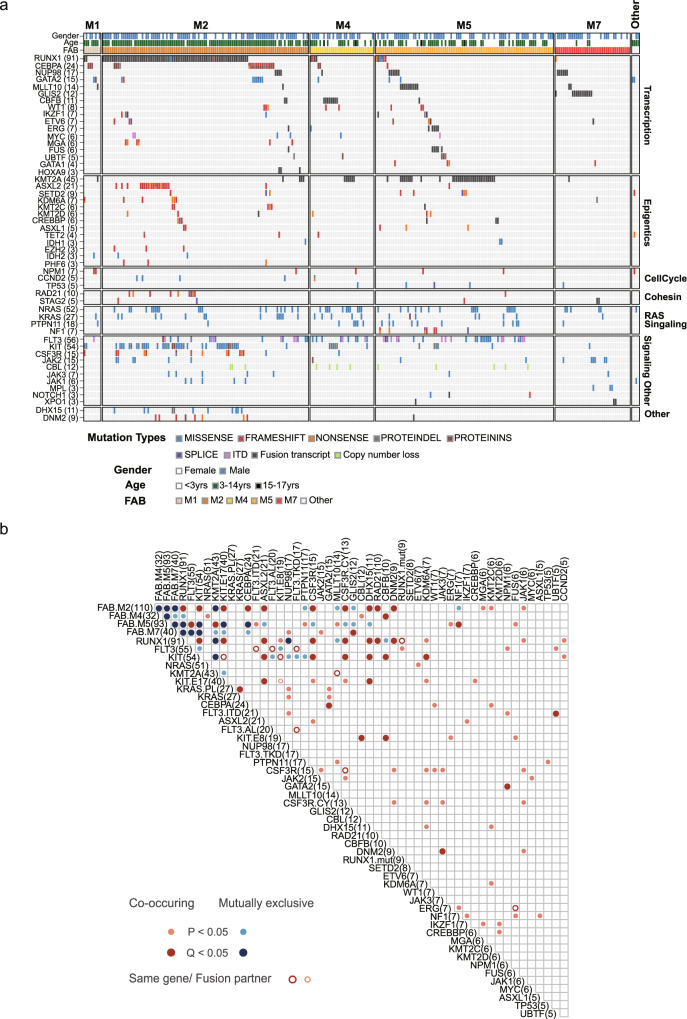
Landscape and correlation of driver genes in Chinese AML. **a** Heat-map displays genes mutated in ≥3 AML cases. Cases are separated into FAB groups (columns), and the genes are grouped into six biological pathways (rows). Colors represent mutation types. **b** Pairwise co-occurrence (red) and mutual exclusive (blue) correlations between driver genes (two-sided Fisher’s exact test). The correlations are labeled with hollow circle if the pairs of mutations/genes were from a same gene or fusion.

We further integrated different variant types to analyze the genomic landscape within Chinese pediatric AML. In addition to the above-described fusions and sequence mutations, we also analyzed the *CBL* exon 8/9 deletion identified in RNA-seq analysis (Methods), which was recurrently deleted in AML^5,26^. This focal deletion was identified in 12 cases in our cohort (4.1%) (Supplementary Data 9 and Supplementary Fig. 5), comparable to the TARGET cohort (4.2% vs 3.6% for patients <15 years, *P* = 0.704). Overall, driver mutations identified in 93 genes were grouped into six pathways (Fig. 3a and Supplementary Data 10). The most frequently mutated pathways in AML were transcription regulation, epigenetics, and RAS signaling, which were mutated in 69.2%, 36.6%, and 32.2% of patients, respectively. Mutations activating other signaling pathways, including JAK-STAT and others, were detected in a total of 50.7% patients. Notably, the RAS signaling pathway was significantly less frequently mutated in Chinese patients (32.2% vs. 49.0%, *P* < 0.001), consistent with the low mutation frequency observed for individual genes in the RAS pathway.

We next investigated the pairwise relationships of driver mutations detected in Chinese AML patients. A total of 150 pairs of genes, mutation hotspots, or FAB groups (143 after excluding pairs between fusion partners or different domains within a single gene) were found to be significantly concurrently or exclusively mutated (*P* < 0.05, two-sided Fisher’s exact test) (Fig. 3b and Supplementary Data 11). Through this analysis, we established several associations in AML, including the co-mutation of *CSF3R* and *KIT*, *ASXL2* and *KIT*, *DHX15* and *KIT*, and *DNM2* and *JAK3*, among others. The observation of these associations that have not previously been described could be only partly explained by the higher mutation frequency of these genes among Chinese AML patients, as completely different mutation associations were also discovered in different populations. For example, *CSF3R* was found to be significantly co-mutated with *KIT* in Chinese cohort, but reportedly co-occurs with *CEBPA* in the Western population^4^. Similarly, *NF1* mutations was found to be significantly associated with the FAB M5 group in Chinese patients (*P* < 0.001) but were previously found to be co-mutated with *CBFB-MYH11* fusion in the M4 group^6^.

### Impact of driver genomic alterations on clinical outcomes

**Fig. 4:**
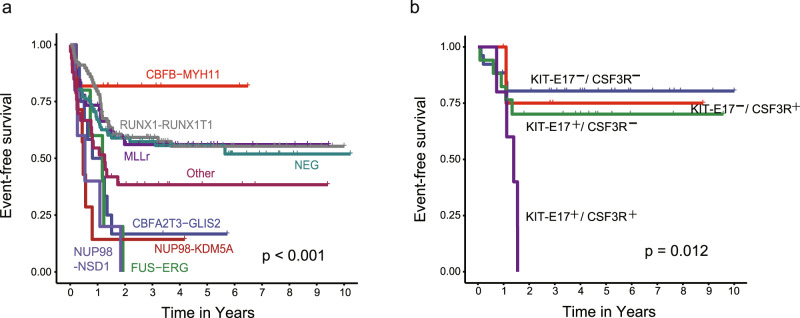
Event-free survival of Chinese pediatric AML. **a** Kaplan-Meier estimate of EFS of Chinese pediatric AML patients diagnosed and treated at SCMC. A total of 288 cases with sufficient follow-up information were included in this analysis. Patients were grouped by the fusions, including *CBFB-MYH11*, 11 cases; *RUNX1-RUNX1T1*, 78; *KMT2A* rearranged (MLLr), 45; *CBFA2T3-GLIS2*, 12; *NUP98-KMD5A*, 7; *NUP98-NSD1*, 5; *FUS-ERG*, 5; other fusions, 33 and fusion negative, 92 (log-rank test, *P* = 4.963E − 05). **b** *RUNX1-RUNX1T1*-positive patients who reached complete remission after the 1^st^ cycle of induction therapy, and had both *CSF3R* and *KIT*-E17 mutations (5 cases), showed inferior prognosis compared to patients carrying only *CSF3R* (*n* = 5) or *KIT*-E17 mutation (*n* = 17) or none of these two mutations (*n* = 26) (log-rank test).

Survival analysis revealed driver genomic aberrations associated with patient prognosis (Supplementary Data 12 and Fig. 4a). Consistent with previous reports, we found that the *CBFB-MYH11* fusion was associated with favorable outcome, while *NUP98-KDM5A/NSD1*, *FUS-ERG*, and *CBFA2T2-GLIS2* were associated with unfavorable prognosis^7,11,12,27–30^. On the other hand, we noticed that patients with *RUNX1-RUNX1T1* fusion and *KMT2A* rearrangements exhibited similar and intermediate 5-year event-free survival (EFS) rates of 55.3% (CI 44.8–68.2%) and 56.1% (CI 42.9–73.3%), respectively. Regarding the mutations, we found that mutations in *CEBPA*, *NPM1*, and *GATA2* were associated with favorable prognosis, while mutations in *RUNX1* and *FLT3* ITD were associated with worse prognosis, which is consistent with previous findings in Western cohort^31–38^. Patients carrying the driver genomic aberrations above showed comparable prognosis in Chinese and Western cohort (Supplementary Results and Supplementary Fig. 6). Besides these previously established associations, we also found that patients carrying *UBTF* mutations showed worse prognosis compared to wild-type (Supplementary Fig. 7a). Furthermore, the impact of these driver variants on patient prognosis was further influenced by co-occurrence relationships. Patients carrying *FLT3* variants together with *UBTF* mutation, *RUNX1* mutation, or *NUP98* rearrangements exhibited worse prognosis compared to patients carrying *FLT3* variants alone (Supplementary Fig. 7b–d). On the other hand, patients with *FLT3* ITD and *NPM1* mutation together showed good outcomes in our current analysis (Supplementary Fig. 7e). We also applied univariate and multivariate Cox regression analysis in this study (Supplementary Data 13a, b). While univariate Cox regression revealed consistent results as above, multivariate Cox regression showed that *CBFA2T3-GLIS2*, *FUS-ERG*, *NUP98* rearrangements, *FLT3* ITD and *RUNX1* mutation were independently associated with adverse prognosis, while *GATA2* was independently associated with favorable outcome, with *P* < 0.05. *UBTF* and *CEBPA* mutation were not included in the multivariate Cox regression model due to the significant co-mutation with *FLT3* ITD and *GATA2*, respectively. Furthermore, we combined the above five genomic factors associated with worse outcome into high risk genotype and carried out the multivariate Cox regression analysis again, together with CR1 status and *GATA2*. Results showed that the combined high-risk genotype was independent risk factor significantly associated inferior prognosis (*P* < 0.001).

Within our cohort, 78 patients carried at least one of the above-described alterations associated with favorable (*n* = 28) or poor outcome (*n* = 50). We next examined whether the remaining patients showed any other clinic relevant alterations. To this end, we focused on the patients who lacked any aberrations having well-established associations with prognosis. In addition to the 78 patients above, 24 were further excluded because they carried mutations in *TP53* (*n* = 3) or *ASXL1* (*n* = 1), *DEK-NUP214* fusion (*n* = 1), or confirmed chromosomal abnormality (*n* = 19, including complex karyotype, monosomal karyotype, −7, −17, del(5q)). We found that for the remaining patients, treatment response after the first cycle of induction therapy was among the factors most significantly associated with patient prognosis (*P* < 0.001, Supplementary Fig. 8a). Patients who did not achieve complete remission after one cycle of induction (CR1) showed adverse outcomes, similar to patients carrying genomic variants associated with poor outcome (*P* = 0.155, Supplementary Fig. 8b). On the other hand, while patients with CR1 showed relatively good outcomes, these patients had a worse prognosis compared to patients carrying variants related to favorable prognosis (*P* = 0.081) (Supplementary Fig. 8c), indicating a mixture of patients within this CR1 group.

We next analyzed the association between genomic aberrations and prognosis within each fusion subtype of the 129 CR1 patients, including *RUNX1-RUNX1T1* (*n* = 53), *KMT2A* rearrangement (*n* = 30), cases with other fusions (*n* = 12), and fusion-negative cases (*n* = 34). We found that *CSF3R* (*P* = 0.038) and *KIT*-E17 (*P* = 0.064) mutations in *RUNX1-RUNX1T1* patients were associated with adverse prognosis (Supplementary Fig. 9). Notably, *CSF3R* and *KIT* exon17 mutations significantly co-occurred within the *RUNX1-RUNX1T1* fusion subtype. Indeed, CR1 patients within the *RUNX1-RUNX1T1* fusion subtype who carried both *CSF3R* and *KIT* exon17 mutations exhibited a significantly worse prognosis compared to patients carrying either one or none of these two mutations (*P* = 0.012) (Fig. 4b).

### Revised risk classification for Chinese pediatric AML patients

**Fig. 5:**
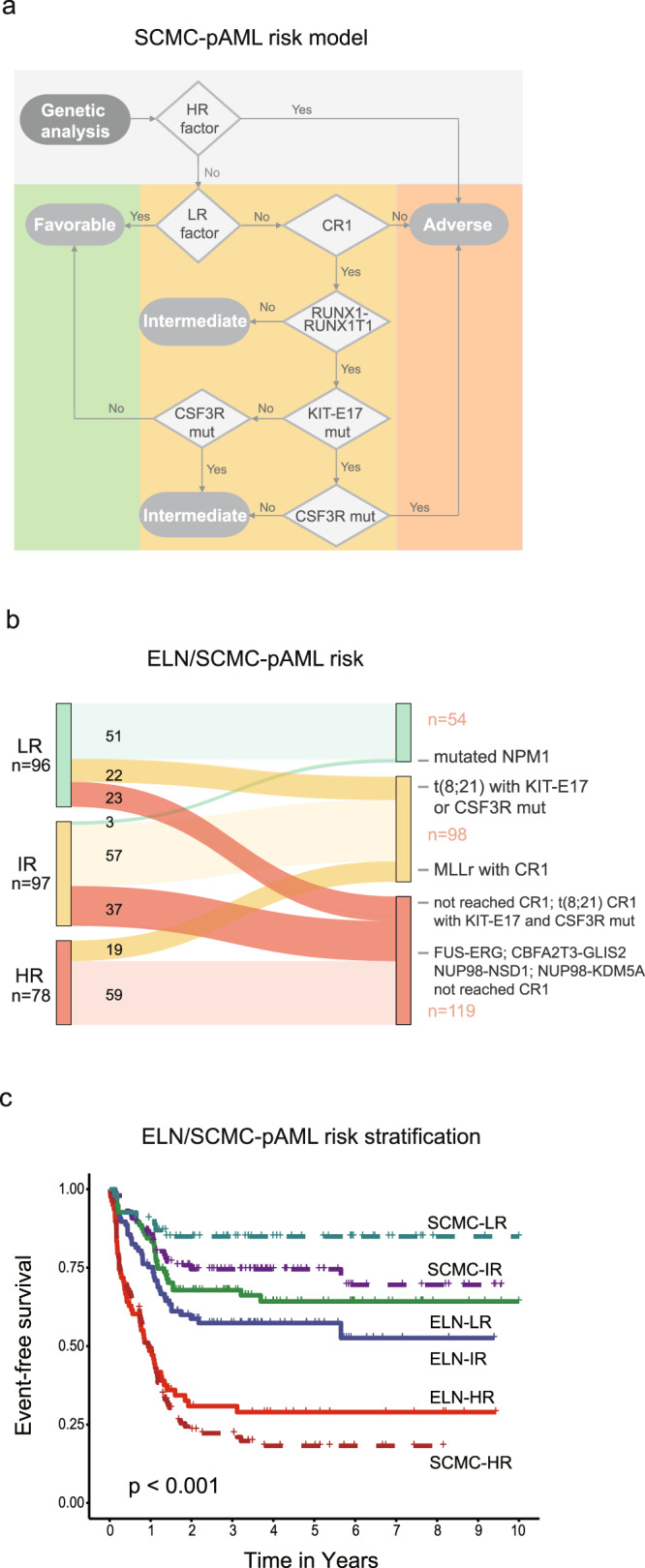
Revised risk stratification model for Chinese pediatric AML. **a** Flow chart of the revised risk stratification model, SCMC-pAML, for Chinese pediatric AML. The model was constructed on top of the European LeukemiaNet (ELN) model, revised to include specific clinically relevant driver alterations established in Chinese pediatric AML. **b** The revised SCMC-pAML model was used to re-stratify patients into risk groups. Risk groups from the SCMC-pAML model and ELN model are shown on the right and left, respectively. Colors indicate different risk factors, and line width is proportional to the number of cases. The genomic alterations leading to re-stratification are labeled on the right. Numbers in the plot indicate the number of cases in each category. A total of 271 cases with sufficient follow-up information were re-stratified using the SCMC-pAML model and included in survival analysis. **c** Event-free survival rate estimates for risk groups stratified using the SCMC-pAML (dashed lines) and ELN model (solid lines). The SCMC-pAML model significantly increased the power of risk stratification of Chinese pediatric AML patients, with low- and intermediate-risk patients showing more favorable outcome, and high-risk patients showing worse outcome (log-rank test, *P* = 2.859E−26).

We next revised the European LeukemiaNet (ELN) genetic risk classification^32^ model based on the clinical relevance established in this study. The proposed SCMC-pAML model (Fig. 5a and Supplementary Data 14) featured adjusted risk classification of several genetic aberrations. For example, *FUS-ERG*, *CBFA2T3-GLIS2*, *NUP98*-*KDM5A* and *NUP98*-*NSD1* were grouped into the high-risk category (HR), while *KMT2A* rearrangements were classified into an intermediate-risk group (IR). Importantly, patients with the *RUNX1-RUNX1T1* fusion, which has been associated with favorable prognosis in previous models, were further subdivided based on CR1 status and subsequently acquired mutations, including *CSF3R* and *KIT*-E17 mutations. Compared to the ELN model, the SCMC-pAML model identified more HR patients and less low-risk (LR) patients, along with a similar number of IR patients (Fig. 5b): with the SCMC-pAML model vs. the ELN model, the percentages of LR, IR, and HR, respectively, were 19.9% vs. 35.4%, 36.2% vs. 35.8%, and 43.9% vs. 28.8% (two-sided Fisher’s exact test, *P* < 0.001). Although the risk groups classified by both models revealed significant differences in patients’ prognoses, the LR and IR groups classified with SCMC-pAML exhibited more favorable outcomes (Fig. 5c), with 5-year EFS rates of 84.9% (95% CI 75.8–95.1%) and 74.5% (95% CI 66.2–83.9%), respectively. These rates were significantly higher than in the LR (64.2%, 95% CI 54.8–75.4%, log-rank test, *P* = 0.02) and IR (57.3%, 95% CI 48.1–68.4%, log-rank test, *P* = 0.01) groups stratified with the ELN model. On the other hand, the SCMC-pAML HR group showed a trend of worse prognosis compared to the ELN HR group, with 5-year EFS rates of 18.2% (95% CI 12.0–27.7%) vs. 30.9% (95% CI 21.8–43.7%), without a statistical difference (log-rank test, *P* = 0.40). Furthermore, being stratified into the HR group with the SCMC-pAML model was identified as an independent risk factor in a multivariable cox model, with a significantly increased risk of adverse event in SCMC-pAML HR patients (hazard ratio 7.7, 95% CI 3.5–17.1, *P* < 0.001) (Supplementary Data 15).

## Discussion

Here we present the comprehensive genomic landscape of driver variants in a large cohort of Chinese pediatric AML. Through comparison to the driver landscape defined in Western population, we first delineated a landscape of driver variants in Chinese AML, recapitulating previously findings. Furthermore, our analyses revealed substantial distinctions between the two cohorts, including less frequent mutations in the RAS signaling pathway and higher mutation rates in *KIT* and *CSF3R*, among others. These differences between Chinese and Western patients could be replicated by analyzing genomic aberrations reported by two independent studies representing Japanese pediatric AML^39^ and French^40^ pediatric core binding factor AML (Supplementary Fig. 10 and Supplementary Data 16). Moreover, by examining the co-mutations between pair of genes, we demonstrated that the cohorts also differed in the co-occurrence of gene pairs. These results show an unexpected difference in genomic aberrations between different populations with a same clinically defined disease. With the causal yet unveiled, the observed difference might be related to genetic or environmental factors. As people start to appreciate, mutagenesis could be affected by local structures within the genome, including DNA accessibility and gene transcription, among others^41^. The genetic polymorphism associated with these genomic features might contribute to the different mutation frequencies observed among populations.

Importantly, the distinct driver variants profile of Chinese AML was relevant to patient outcomes. For example, *CSF3R* mutation reportedly co-occurs with *CEBPA* mutation in a Western cohort. However, in our Chinese cohort, we found significant co-occurrence between *CSF3R* and *KIT*. Moreover, this co-occurrence, specifically between *CSF3R* and mutation in exon 17 of *KIT*, was associated with worse event-free survival among CR1 patients of the *RUNX1-RUNX1T1* fusion subtype. Besides the five patients reported in current study, we identified additional two *RUNX1-RUNX1T1* positive patients diagnosed and treated at SCMC who reached CR1 and carrying both *CSF3R* and *KIT-*E*17* mutations. The first patient relapsed after 8 months from diagnosis and was detected with CSF3R L780fs (VAF = 0.306), KIT N822K (VAF = 0.165), and KIT D820G (VAF = 0.012) mutations. The second patient was enrolled into RNA-seq analysis during relapse. We detected CSF3R T618I (VAF = 0.459) and KIT D816V (VAF = 0.118) in the relapsed sample. Both mutations could be detected at diagnosis with target sequencing, with a low VAF for both mutations (CSF3R T618I VAF = 0.007 (30/4008) and KIT D816V VAF = 0.012 (75/6001)). Both patients relapsed within one year from diagnosis, consistent with the poor prognosis observed in current cohort. All 7 patients (including the two described above) in this category reached CR1 during the treatment, suggesting the *CSF3R* and *KIT-*E*17* mutations were not resistant to current chemotherapy but indicating a higher risk for relapse. The mechanism remains further investigation.

Consistent with EFS analysis, results of the driver genomic aberrations’ impact on patient prognosis were observed from analyzing overall survival (OS) of patients (Supplementary Fig. 11). Based on these observations, we proposed the SCMC-pAML model to more precisely reflect genomic aberration-based risk classification in Chinese pediatric AML patients. The refined model significantly improved the risk stratification, as shown in the multivariable cox model. In our current analysis, the SCMC-pAML model could influence and refine the clinical risk stratification of 35.6% of patients (104 out of 292), including a total of 60 patients re-stratified into the HR group. The performance of this revised model requires further validation with multi-center clinical studies. However, its power to precisely classify HR patients can already potentially guide the development of specific treatments for this patient group, as a next step of precision medicine.

In addition to the distinct profile of mutation frequencies, our analyses also identified several potentially drivers, including an in-frame fusion involving *XPO1*. This fusion was previously reported in one pediatric B-ALL from Children’s Oncology Group^42^ and one pediatric AML from Swedish group^43^. In current study, we showed *XPO1-TNRC18* fusion was recurrent and enriched in pediatric AML-M7. Patients carrying the *XPO1* fusion exhibited no other oncogenic fusion, indicating that this fusion was likely the driver in these tumors. Moreover, we have observed more cases carrying this in-frame fusion through real-time genomic analysis of AML patients diagnosed at SCMC, and all presently discovered cases belong to the M7 subtype. Notably, an inhibitor targeting *XPO1* is available, suggesting a potential treatment target in pediatric AML. The E571K mutation was identified as a hotspot mutation in *XPO1* in multiple types of cancer^19–22^. This mutation located in the nuclear export signal (NES)-binding groove of XPO1 protein and was suggested to affect the binding preferences of XPO1 for nuclear export protein and RNA^19,22,44,45^, and play an oncogenic role in cancer. On the other hand, the role of *TNRC18* in cancer was still unclear. The XPO1-TNRC18 fusion protein contains both the wild type NES-binding groove of XPO1 and the BAH (Bromo Adjacent Homology) domain of TNRC18 protein which is associated with transcriptional silencing and chromatin remodeling through recognizing histone modification or protein-protein interaction^46,47^. The fuse of these domains might result in mislocalization of chromatin modification proteins in the cell. The molecular mechanism of this fusion in leukemia remains further investigated.

By analyzing transcriptome sequencing data of AML tumor cells, we systematically analyzed both fusions and sequence mutations in a single experiment. We demonstrated that the mutation analysis from RNA-seq was highly consistent in driver mutation detection, as compared to DNA-based analysis, including WES and WGS. Furthermore, the RNA-seq-based mutation analysis could achieve even higher coverage (>1000×) than WES for oncogenes. This was due to the nature of high transcription of these genes, further increasing the sensitivity of analyzing subclonal mutations in these genes, which are common in leukemias^48–50^. On the other hand, this approach would have limitations for analyzing genes that were not transcribed or were down-regulated in transcription, as well as in analyzing copy number aberrations. With these limitations, our analysis might have underestimated the occurrence of tumor suppressor genes.

In summary, here we characterized the comprehensive genomic landscape of Chinese pediatric AML. Our results unveiled a clinically relevant mutation profile that was distinct from that of the Western cohort, in terms of both mutation frequency and patterns of mutation co-occurrence. These findings further elucidate the complexity of pediatric AML and highlight the importance of considering ethnic background when establishing risk stratification for clinical management in the era of precision medicine.

## Methods

### Patient samples

Bone marrow samples were obtained from 292 patients diagnosed as AML through 2001–2018 in the Department of Hematology and Oncology, Shanghai Children’s Medical Center (SCMC). The research was approved by the Ethics Committee at SCMC. Informed written consents were obtained from parents for all patients.

### Treatment

Patients enrolled in AML-SCMC-2009 protocol were treated with ten courses of chemotherapy in three treatment phases. During induction treatment standard 3 + 7 regimen was given in the first course, including daunorubicin (DNR) 40 mg/m^2^ per day on days 1–3, cytarabine (Ara-c)100 mg/m^2^ every 12 h on days 1–7, and etoposide (VP-16) 100 mg/m^2^ per day on days 5–7. After evaluation at the end of the first induction, patients were treated with (Group A) or without (Group B) anthracycline as following treatment. During the second induction, patients received mitoxantrone 10 mg/m^2^ on days 1–3 (Group A) or homoharringtonine (HHT) (Group B) instead of DNR as in the first induction. In consolidation phase four courses of high-dose Ara-c at dosage 3.0 g/m^2^ for six doses with 12 h interval coupled with DNR 40 mg/m^2^(hAD), or mitoxantrone 10 mg/m^2^ (hAM), or VP-16 100 mg/m^2^ (hAE) for 2 days in Group A, or HHT 3 mg/m^2^ for 5 consecutive days (hAH) to substitute DNR and mitoxantrone in Group B. During maintenance therapy, patients were given mercaptopurine 75 mg/m^2^ every day and Ara-C 75 mg/m^2^ every 12 h for 7 days in Group A or HHT 3 mg/m^2^ every day for 9 days and Ara-C 75 mg/m^2^ every 12 h for 7 days in Group B for 4 cycles respectively. Ten times (for FAB M4 and M5) or four times triple intrathecal therapy with methotrexate, cytarabine, and hydrocortisone at an age-adjusted dose was exploited as central nervous system prophylaxis. Patients who failed to achieve complete remission by the first induction therapy was the indication for hematopoietic stem cell transplantation.

### Transcriptome sequencing (RNA-seq) and analysis

Total RNA was extracted from frozen bone marrow cells of AML samples with TRIzol, and checked for RIN (>6) to inspect RNA integrity. RNA-seq experiments were performed according to manufacturer’s instruction with total RNA-seq or mRNA-seq protocol (Supplementary Data 1). For strand-specific sequencing, cDNA libraries were prepared with Illumina TruSeq Stranded Total RNA Kit. The purified cDNA libraries were sequenced on the Illumina HiSeq 2000, HiSeq X Ten or NovaSeq 6000 system (Illumina, United States) with PE 150 bp. RNA-seq data was aligned to human genome version GRCh37-lite with STAR^51^ and mark duplicate with Picard. Single nucleotide variants (SNVs) and indels were detected with MuTect2^52^ and Rnaindel^53^. Variants were annotated with VEP^54^ and filtered for variants that were likely somatic and/or associated with cancer with in house code as described below. Fusions were analyzed with CICERO^25^ and FusionCatcher^55^. Internal tandem duplication (ITD) was analyzed with CICERO^5,56^.

### SNV and Indel analysis

Following steps were applied to identify potential somatic and/or cancer-associated variants. First, variants were excluded if: (1) in Ig/TCR region; (2) mutant reads <3 or depth <8; (3) frequency in 1000 Genomes database >0.001; (4) mutation type did not match missense variant, stop gained, inframe insertion/deletion, frameshift, splice acceptor/donor, stop lost, start lost, transcript amplification, protein altering variant, splice region variant or coding sequence variant; (5) in variant artifact list constructed with the 292 cases analyzed in this study. The variants were further grouped into 3 levels. L1 variants met any of the following criteria: (1) Recurrent (reported in ≥3 tumors in the COSMIC database^57^) and variant allele frequency (vaf) ≥0.1; (2) Previously reported in pediatric cancer genomic projects including PCGP and TARGET; (3) Loss of function variants (frameshift, splicing and stop gained) in known tumor suppressor genes; (4) “Pathogenic” or “Likely Pathogenic” in ClinVar; (5) vaf > 0.1, ‘deleterious’ and ‘possibly_damaging’ in SIFT and PolyPhen analysis, in COSMIC cancer gene census and absent from 1000 Genomes database. Variants did not meet above criteria would be grouped into L2, if: Recurrent (reported in ≥3 tumors in the COSMIC database) and vaf ≤ 0.1 or not in 1000 Genomes database. All other variants were grouped into L3. L1 and L2 mutations were considered to be putative somatic and/or cancer associated. Variants were manually curated to exclude artifacts. Furthermore, nonsynonymous variants detected in the genes known to be recurrently mutated in AML (including *KIT*, *CEBPA*, *WT1* among others) were manually curated.

### Driver mutation analysis

PeCanPIE^24^ and MutClan (Cui B., Sun H., Rao J., Zhao S., Wang H., Liu T., Wang R., Shen S. Liu Y., Manuscript in preparation) analysis were applied to identify driver mutations. MutClan was designed to identify mutations appeared in a cluster of previously reported somatic mutations. A total of 6,975,733 published somatic mutations in 983 pediatric tumors collected from St. Jude Cloud^58^ were used to construct the mutation cluster background. A mutation would be considered as potential driver if classified as gold in PeCanPIE analysis or significantly located in mutation cluster (fdr *q*-value < 0.05). For mutations in TARGET cohort, we included the multi-platform verified somatic genomic variants reported in the Bolouri et al (Nature Medicine 2018), including both discovery and validation dataset. PeCanPIE analysis was applied to TARGET mutations and only mutations classified with a medal were included in the analysis. Results were manually reviewed for each mutation and visualized using ProteinPaint^59^.

### Evaluation of driver mutation detection with RNA-seq

Ten ALL diagnosis samples with matched RNA-seq and WGS data published previously were collected^23^. RNA-seq data were analyzed for SNVs and Indels as described above. SNVs and Indels detected with WGS were collected as a benchmark. PeCanPIE was applied to all mutations and only mutations classified as gold or silver were included in this analysis. For mutations detected specifically in RNA-seq, capture sequencing data from previous published study^23^ were curated whenever available.

### Statistical analysis

Association between mutations was examined using Fisher’s exact test. Kaplan–Meier estimation, log-rank test and Cox regression analysis were applied to the survival analysis. EFS was defined from diagnosis to the first major adverse event, including relapse after remission, failure to achieve remission, death due to any cause, abandonment, development of a second malignancy, or transfer to other hospital. OS was defined from diagnosis to death of any cause. There were 82 patients (28.1%) abandoned treatment by parental decision, due to relapse (*n* = 41, 50%), poor treatment response (*n* = 24, 29.3%), drug resistance (*n* = 14, 17.1%), second tumor (*n* = 2, 2.4%), or unknown reason (*n* = 1, 1.2%). These patients were grouped to event in OS analysis, with the last follow-up date as date of event. Patients lost to follow-up were censored at the date of last known contact.

Analyses were performed using Rstudio v4.1.0.

### Reporting summary

Further information on research design is available in the Nature Research Reporting Summary linked to this article.

## Supplementary information

Supplementary Information

Description of Additional Supplementary Files

Supplementary Data 1

Supplementary Data 2

Supplementary Data 3

Supplementary Data 4

Supplementary Data 5

Supplementary Data 6

Supplementary Data 7

Supplementary Data 8a

Supplementary Data 8b

Supplementary Data 9

Supplementary Data 10

Supplementary Data 11

Supplementary Data 12

Supplementary Data 13a

Supplementary Data 13b

Supplementary Data 14

Supplementary Data 15

Supplementary Data 16

Reporting Summary

## Data Availability

The RNA-seq data generated in this study have been deposited in the Genome Sequence Archive (GSA) for Human of the National Genomics Data Center of China under accession number HRA000789. The data is available for academic use under controlled access in compliance with the regulation of the Ministry of Science and Technology (MOST) of China for the deposit and use of human genomic data. Access can be obtained by contacting members of the Data Access Committee (DAC) Shuhong Shen at shenshuhong@scmc.com.cn or Yu Liu at liuyu@scmc.com.cn and following the application procedure in GSA. For detailed guidance, see GSA-Human_Request_Guide_for_Users [https://ngdc.cncb.ac.cn/gsa-human/document/GSA-Human_Request_Guide_for_Users_us.pdf]. Data will be available immediately once the application was approved. The access to the controlled data will be valid for one year from the date approved. The processed genomic aberrations from this dataset are available within the Supplementary Information files. The publicly available genomic data for TARGET AML are available in the database of Genotypes and Phenotypes (dbGap) under accession number phs000465. The clinical annotations of TARGET AML cohort and verified somatic genomic variants from both discovery and validation dataset are downloaded from Bolouri et al.^4^ and TARGET Data Matrix (https://ocg.cancer.gov/programs/target/data-matrix). The 10 previously published RNA-seq data re-analyzed in this study are available as part in GSA for Human under accession number HRA000119. The mutations from Japanese and French pediatric AML cohorts were obtained from Shiba et al.^39^ and Duployez et al.^40^ respectively. The remaining data are available within the Article or Supplementary Information.
