# Supplementary material for: Distinct genomic landscape of Chinese pediatric acute myeloid leukemia impacts clinical risk classification
Source: Nat Commun. 2022 Mar 28;13:1640. doi: 10.1038/s41467-022-29336-y (PMC8960760; doi:10.1038/s41467-022-29336-y)
Supplement: Supplementary file 1 — Supplementary Information [file 41467_2022_29336_MOESM1_ESM.pdf]

**Supplementary Information for**

# Distinct genomic landscape of Chinese pediatric acute myeloid leukemia impacts clinical risk classification

Table of Contents

Supplementary Results....................................................................................2

Supplementary Figures....................................................................................3

# Supplementary Results

**Comparison of clinical impact of driver genomic aberrations between SCMC and TARGET pediatric AML**

We compared prognosis of Chinese (SCMC cohort) and Western (TARGET cohort) pediatric AML for each driver genomic aberration, including fusions (*KMT2A* rearrangements as a whole, *KMT2A-MLLT3*, *KMT2A-MLLT10*, *RUNX1-RUNX1T1*, *CBFA2T3-GLIS2*, *CBFB-MYH11* and *NUP98-NSD1*) and mutations (*FLT3* mutations and ITDs, *KIT*, *NRAS*, *KRAS*, *CEBPA* and *PTPN11*). Patients with age<15 were included in this analysis (n=498 in TARGET and n=288 in SCMC). No statistically significant difference was observed between the two cohorts, indicating a consistent clinic impact for these shared driver aberrations between Chinese and Western cohort (Supplementary Figure 6a). We further checked patients carrying *KMT2A* rearrangements with different partners. As shown in Supplementary Figure 6b-c, pediatric AML carrying *KMT2A-MLLT3* (*MLL-AF9*) fusion showed a statistically significant better prognosis (event-free survival, *P*=0.007) as compared to other *KMT2A* rearranged AML in TARGET cohort. A similar trend was observed in SCMC cohort without a statistic significance (*P*=0.184). Meanwhile, no significant difference of prognosis was observed between SCMC and TARGET cohort carrying either *KMT2A-MLLT3* (*P*=0.565) or other *KMT2A* rearrangements (*P*=0.174). However, we noticed that SCMC patients carrying *KMT2A* rearrangements other than *KMT2A-MLLT3* showed a trend of better prognosis compared to TARGET cohort. This might because of different fusion partners in *KMT2A* rearrangements between the two cohorts. As compared to TARGET cohort, SCMC cohort had more *KMT2A-SEPT6* (6.98% in SCMC vs 2.06% in TARGET) and *KMT2A-AFF1* (6.98% vs 2.06%) fusions and less *KMT2A-MLLT4* (2.33% vs 8.25%) fusion (Supplementary Figure 6d).

## Supplementary Figures

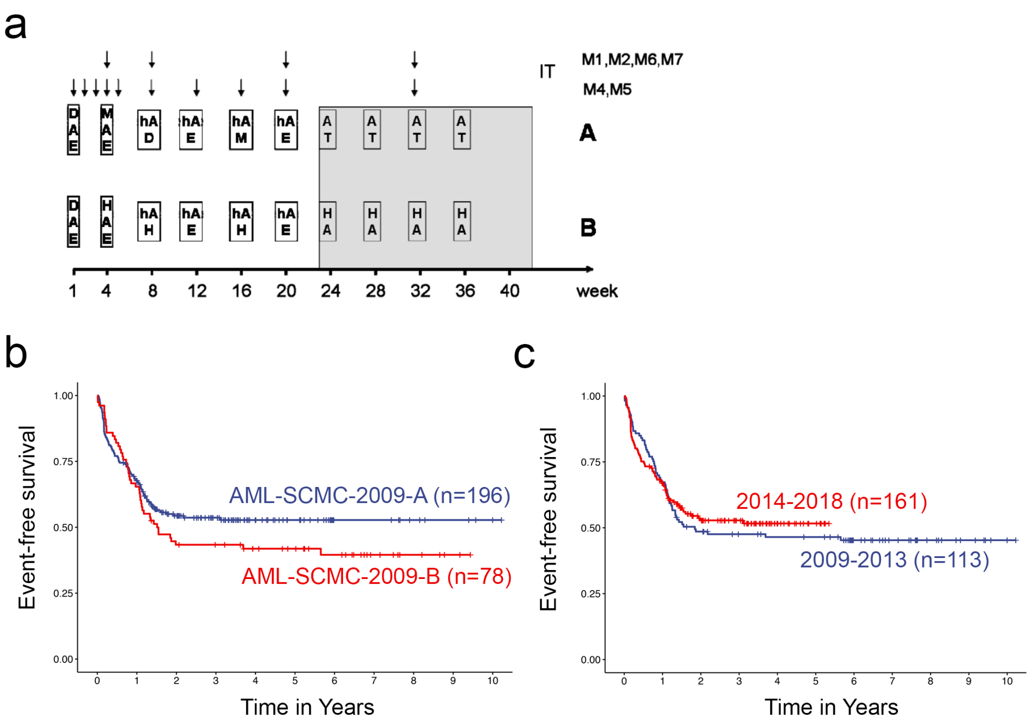


**Supplementary Figure 1. Treatment protocol of pediatric AML patients enrolled in the study.** (a) Schema of AML-SCMC-2009 protocol (Methods). IT, intrathecal therapy. (b) Kaplan-Meier estimate of event-free survival between patients treated on AML-SCMC-2009-A and AML-SCMC-2009-B (*P*=0.207). (c) Kaplan-Meier estimate of event-free survival between patients treated on AML-SCMC-2009 protocol during 2009-2013 and 2014-2018 (*P*=0.655). *P* value was calculated with log-rank test for (b) and (c).

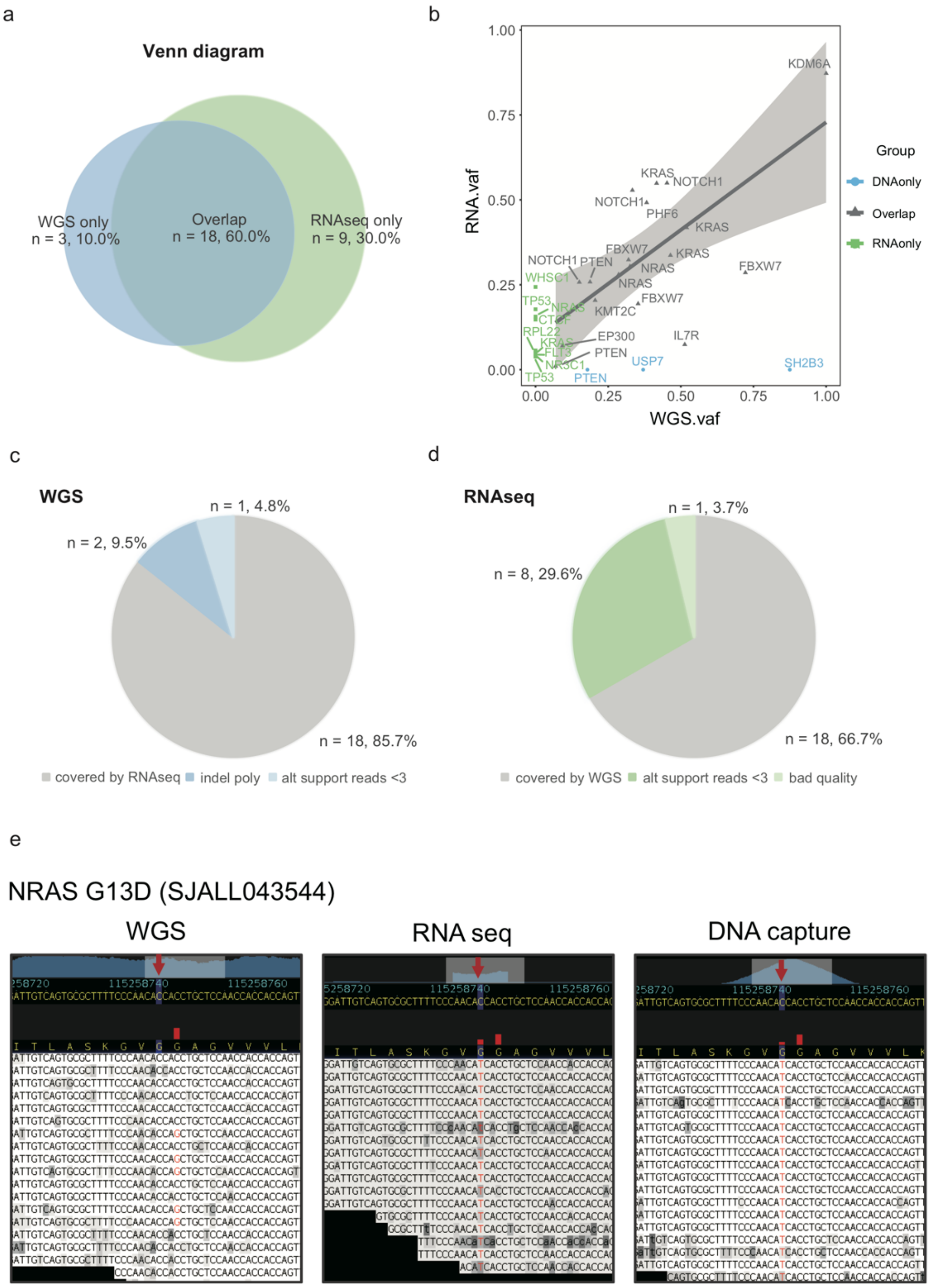


**Supplementary Figure 2. Evaluation and validation of driver mutations analyzed from RNA-seq.** (**a**) Potential driver mutations called from matched whole genome sequencing (WGS) and RNA-seq collected from 10 diagnosis ALL cases were compared and showed in Venn diagram. (**b**) Variant allele frequency (VAF) for each mutation detected in WGS (x-axis) and RNA-seq (y-axis) was shown in scatter plot. Mutations detected in RNA-seq only were labeled in green and aligned to the left of the plot, while mutations detected in WGS only were showed in blue and aligned to the

bottom of the plot. Grey shading represented 95% confidence interval in the linear regression of RNA-seq VAF and WGS VAF of mutations detected in both experiments. (**c**) Breakdown of mutations detected in WGS was shown with pie charts. Grey indicated mutations detected by both WGS and RNA-seq (n = 18); dark blue indicated mutations detected in WGS but not RNA-seq due to false negative results in indel calls with repeated sequences (n = 2) (indel poly); light blue indicated mutations detected in WGS only due to mutant reads from RNA-seq < 3 (n = 1). (**d**) Breakdown of mutations detected in RNA-seq. Grey, mutations detected in both WGS and RNA-seq (n = 18); dark green, mutations detected in RNA-seq only due to insufficient mutant allele (< 3) in WGS (n = 8); light green, mutations detected in RNA-seq only due to bad sequencing quality in WGS (n = 1). (**e**) Validation of mutations detected in RNA-seq but not WGS by DNA capture sequencing. *NRAS* G13D from one sample, (SJALL043544) was shown as an example. All reads from WGS were wild type (left), while mutant reads (in red) could be detected by both RNA-seq (middle) and DNA capture sequencing (right).

**Supplementary Figure 3. Driver sequence mutations in SCMC and TARGET pediatric AML.** Potential driver sequence mutations detected in Eastern (SCMC) and Western (TARGET) cohort were shown with ProteinPaint. The genes shown in this figure were the same as shown in Figure 2b.

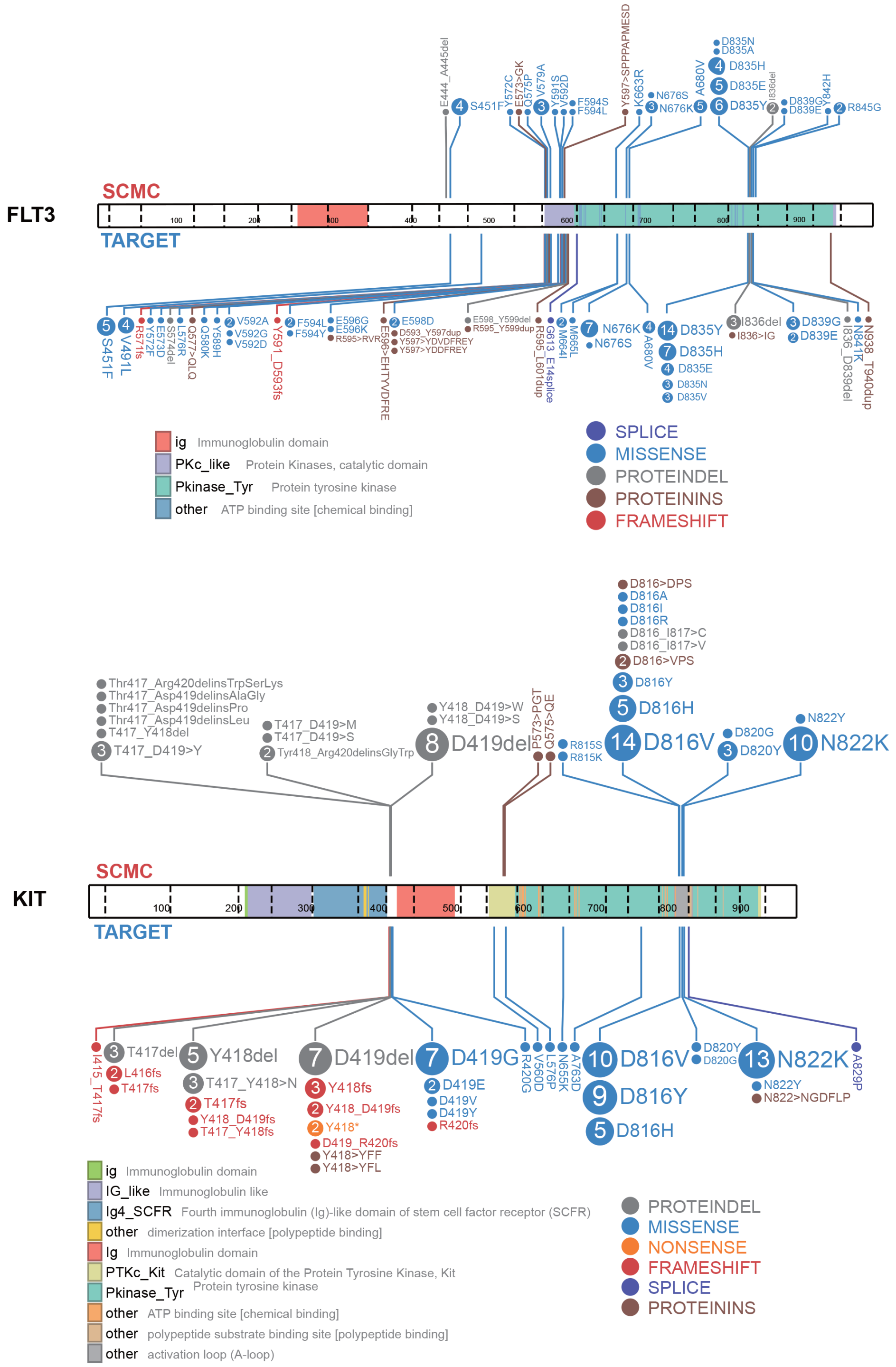

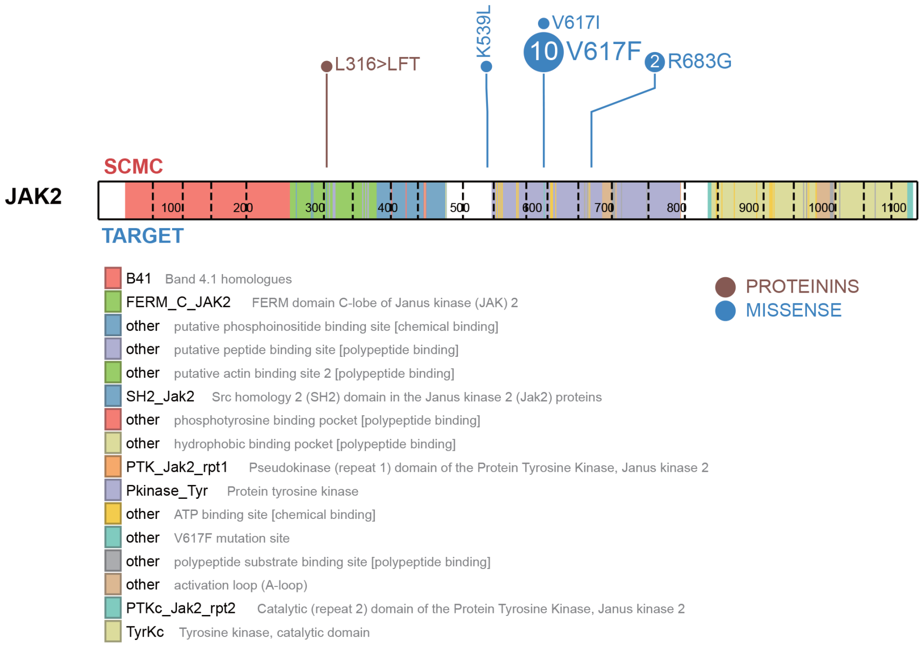

L316>LFT
K539L
V617I
10 V617F
2 R683G
SCMC
JAK2
100
200
300
400
500
600
700
800
900
1000
1100
TARGET
B41 Band 4.1 homologues
FERM_C_JAK2 FERM domain C-lobe of Janus kinase (JAK) 2
other putative phosphoinositide binding site [chemical binding]
other putative peptide binding site [polypeptide binding]
other putative actin binding site 2 [polypeptide binding]
SH2_Jak2 Src homology 2 (SH2) domain in the Janus kinase 2 (Jak2) proteins
other phosphotyrosine binding pocket [polypeptide binding]
other hydrophobic binding pocket [polypeptide binding]
PTK_Jak2_rpt1 Pseudokinase (repeat 1) domain of the Protein Tyrosine Kinase, Janus kinase 2
Pkinase_Tyr Protein tyrosine kinase
other ATP binding site [chemical binding]
other V617F mutation site
other polypeptide substrate binding site [polypeptide binding]
other activation loop (A-loop)
PTKc_Jak2_rpt2 Catalytic (repeat 2) domain of the Protein Tyrosine Kinase, Janus kinase 2
TyrKc Tyrosine kinase, catalytic domain
PROTEININS
MISSENSE


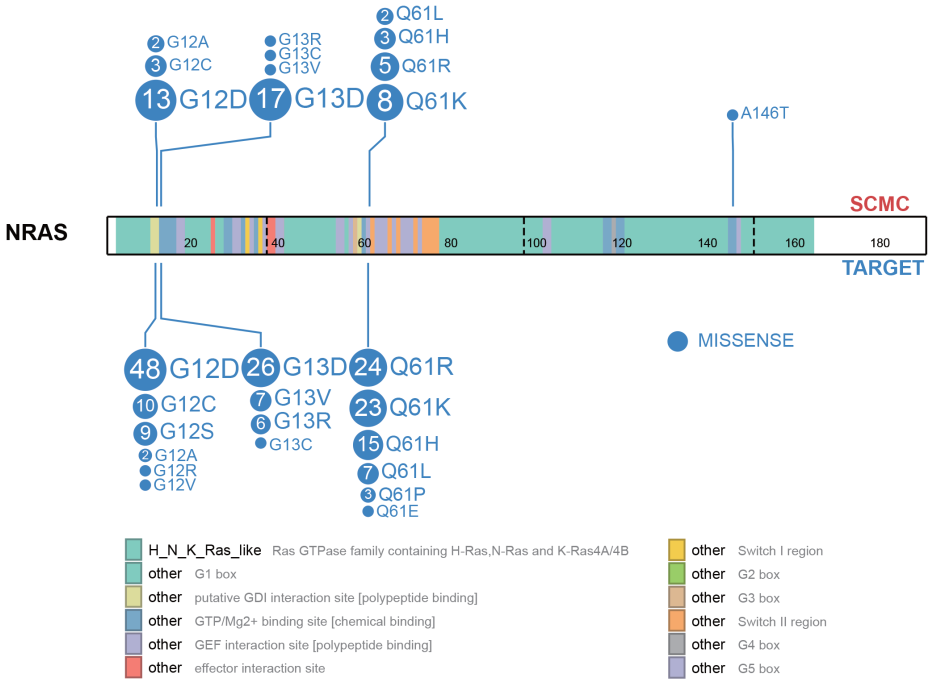

2 G12A
3 G12C
13 G12D
G13R
G13C
G13V
17 G13D
2 Q61L
3 Q61H
5 Q61R
8 Q61K
A146T
NRAS
20
40
60
80
100
120
140
160
180
SCMC
TARGET
48 G12D
10 G12C
9 G12S
2 G12A
G12R
G12V
26 G13D
7 G13V
6 G13R
G13C
24 Q61R
23 Q61K
15 Q61H
7 Q61L
3 Q61P
Q61E
MISSENSE
H_N_K_Ras_like Ras GTPase family containing H-Ras,N-Ras and K-Ras4A/4B
other G1 box
other putative GDI interaction site [polypeptide binding]
other GTP/Mg2+ binding site [chemical binding]
other GEF interaction site [polypeptide binding]
other effector interaction site
other Switch I region
other G2 box
other G3 box
other Switch II region
other G4 box
other G5 box

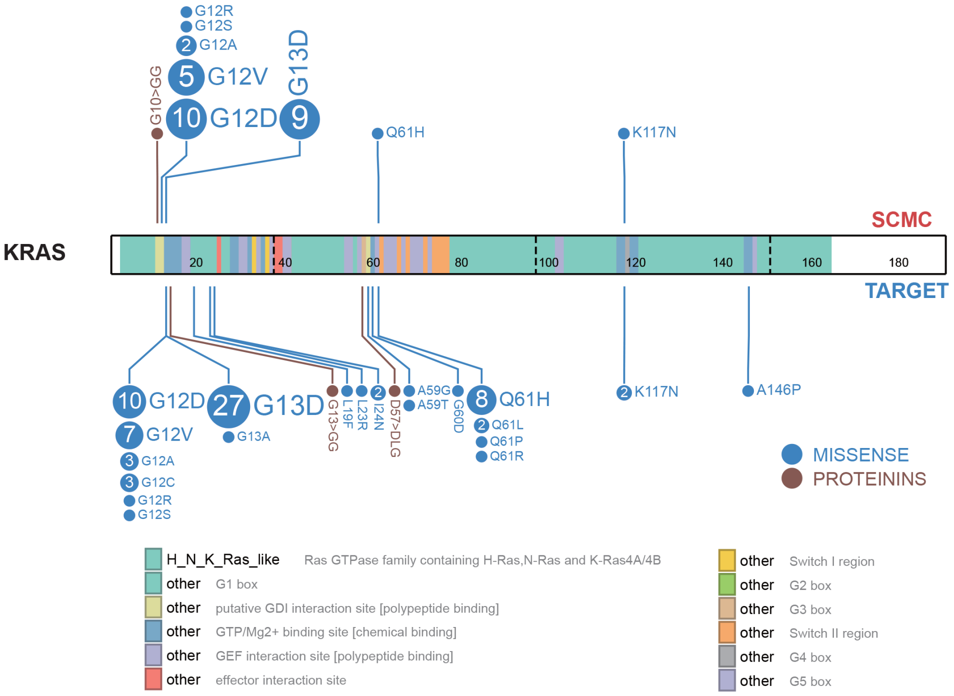
KRAS
SCMC
TARGET
G12R
G12S
2 G12A
5 G12V
10 G12D
9 G13D
G10>GG
Q61H
K117N
20
40
60
80
100
120
140
160
180
10 G12D
27 G13D
7 G12V
3 G12A
3 G12C
G12R
G12S
G13A
G13>GG
L19F
L23R
2 I24N
D57>DLG
A59G
A59T
G60D
8 Q61H
2 Q61L
Q61P
Q61R
2 K117N
A146P
MISSENSE
PROTEININS
H_N_K_Ras_like Ras GTPase family containing H-Ras,N-Ras and K-Ras4A/4B
other G1 box
other putative GDI interaction site [polypeptide binding]
other GTP/Mg2+ binding site [chemical binding]
other GEF interaction site [polypeptide binding]
other effector interaction site
other Switch I region
other G2 box
other G3 box
other Switch II region
other G4 box
other G5 box

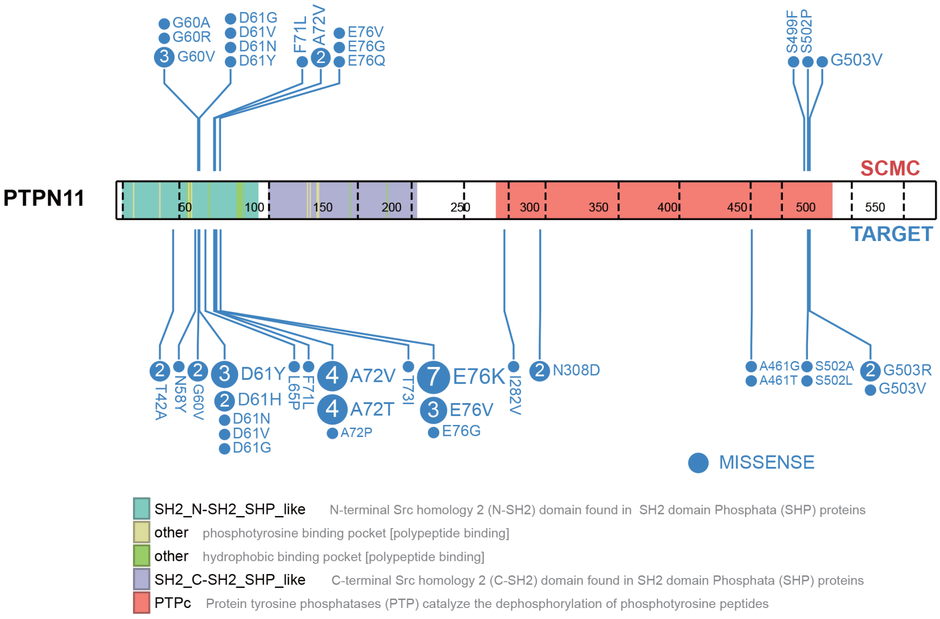
PTPN11
SCMC
TARGET
G60A
G60R
3 G60V
D61G
D61V
D61N
D61Y
F71L
A72V
2
E76V
E76G
E76Q
S499F
S502P
G503V
50
100
150
200
250
300
350
400
450
500
550
2 T42A
N58Y
2 G60V
3 D61Y
2 D61H
D61N
D61V
D61G
L65P
F71L
4 A72V
4 A72T
A72P
T73I
7 E76K
3 E76V
E76G
I282V
2 N308D
A461G
A461T
S502A
S502L
2 G503R
G503V
MISSENSE
SH2_N-SH2_SHP_like N-terminal Src homology 2 (N-SH2) domain found in SH2 domain Phosphata (SHP) proteins
other phosphotyrosine binding pocket [polypeptide binding]
other hydrophobic binding pocket [polypeptide binding]
SH2_C-SH2_SHP_like C-terminal Src homology 2 (C-SH2) domain found in SH2 domain Phosphata (SHP) proteins
PTPc Protein tyrosine phosphatases (PTP) catalyze the dephosphorylation of phosphotyrosine peptides

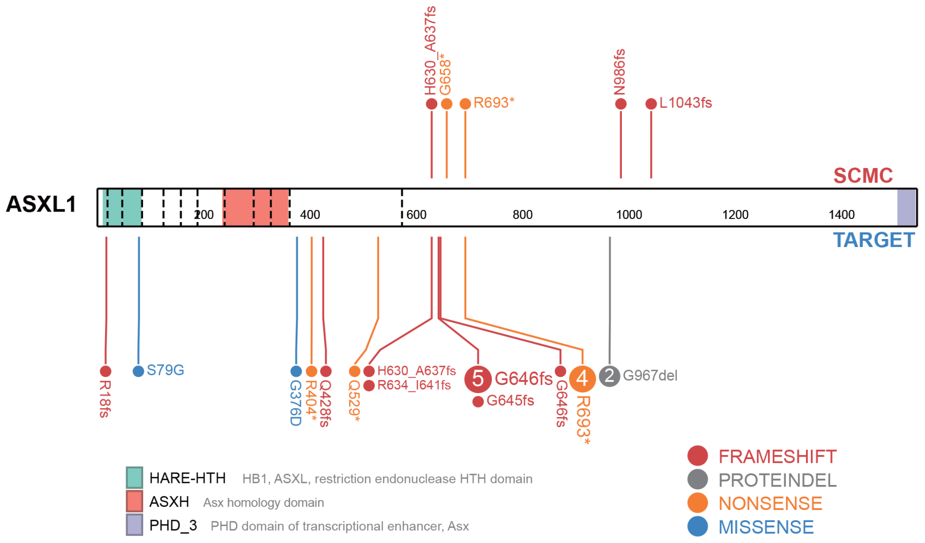
ASXL1
SCMC
TARGET
200
400
600
800
1000
1200
1400
H630_A637fs
G658*
R693*
N986fs
L1043fs
R18fs
S79G
G376D
R404*
Q428fs
Q529*
H630_A637fs
R634_I641fs
5 G646fs
G645fs
G646fs
4 R693*
2 G967del
HARE-HTH HB1, ASXL, restriction endonuclease HTH domain
ASXH Asx homology domain
PHD_3 PHD domain of transcriptional enhancer, Asx
FRAMESHIFT
PROTEINDEL
NONSENSE
MISSENSE

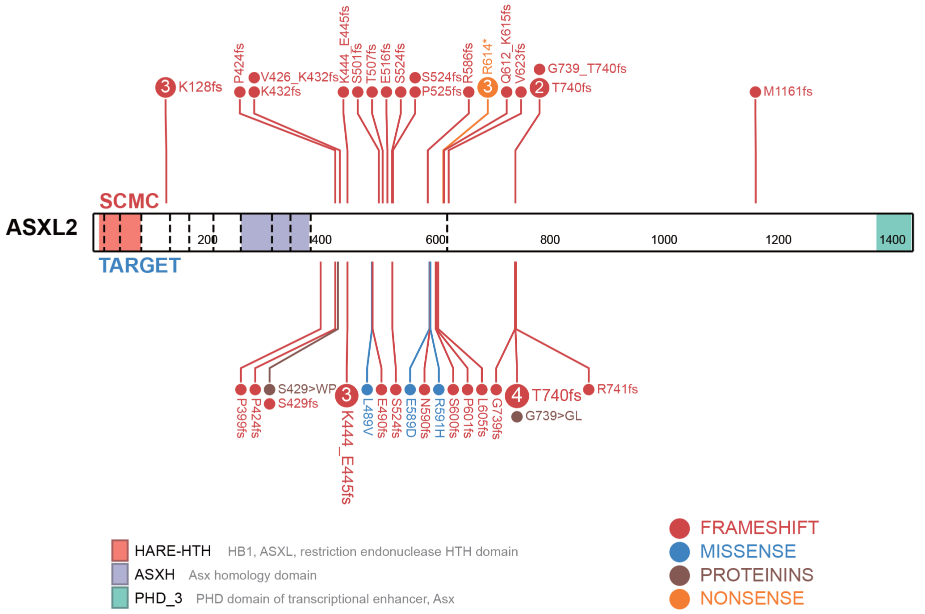
ASXL2
SCMC
TARGET
200
400
600
800
1000
1200
1400
3 K128fs
P424fs
V426_K432fs
K432fs
K444_E445fs
S501fs
T507fs
E516fs
S524fs
S524fs
P525fs
R586fs
3 R614*
Q612_K615fs
V623fs
G739_T740fs
2 T740fs
M1161fs
P399fs
P424fs
S429>WP
S429fs
3 K444_E445fs
L489V
E490fs
S524fs
E589D
N590fs
R591H
S600fs
P601fs
L605fs
G739fs
4 T740fs
G739>GL
R741fs
HARE-HTH HB1, ASXL, restriction endonuclease HTH domain
ASXH Asx homology domain
PHD_3 PHD domain of transcriptional enhancer, Asx
FRAMESHIFT
MISSENSE
PROTEININS
NONSENSE

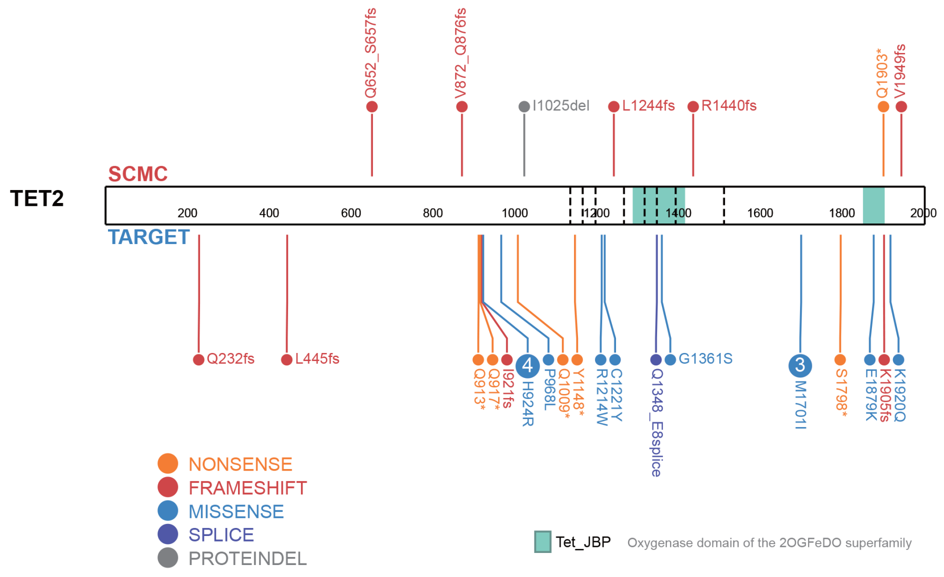
TET2
SCMC
TARGET
Q652_S657fs
V872_Q876fs
I1025del
L1244fs
R1440fs
Q1903*
V1949fs
Q232fs
L445fs
Q913*
Q917*
I921fs
H924R
P968L
Q1009*
Y1148*
R1214W
C1221Y
Q1348_E8splice
G1361S
M1701I
S1798*
E1879K
K1905fs
K1920Q
NONSENSE
FRAMESHIFT
MISSENSE
SPLICE
PROTEINDEL
Tet_JBP
Oxygenase domain of the 2OGFeDO superfamily

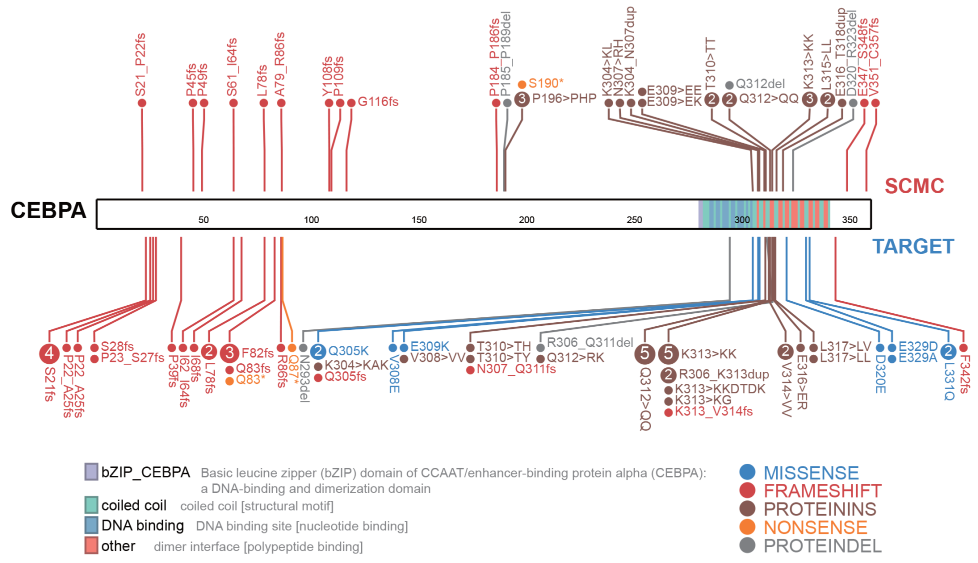
CEBPA
SCMC
TARGET
S21_P22fs
P45fs
P49fs
S61_I64fs
L78fs
A79_R86fs
Y108fs
P109fs
G116fs
P184_P186fs
P185_P189del
S190*
P196>PHP
T310>TT
Q312del
Q312>QQ
K313>KK
L315>LL
S21fs
S28fs
P23_S27fs
F82fs
Q83fs
Q83*
R86fs
Q87*
N293del
Q305K
K304>KAK
Q305fs
E309K
V308>VV
V308E
T310>TH
T310>TY
N307_Q311fs
R306_Q311del
Q312>RK
K313>KK
R306_K313dup
K313>KKDTDK
K313>KG
K313_V314fs
Q312>QQ
V314>VV
E316>ER
L317>LV
L317>LL
D320E
E329D
E329A
L331Q
F342fs
bZIP_CEBPA
Basic leucine zipper (bZIP) domain of CCAAT/enhancer-binding protein alpha (CEBPA): a DNA-binding and dimerization domain
coiled coil
coiled coil [structural motif]
DNA binding
DNA binding site [nucleotide binding]
other
dimer interface [polypeptide binding]
MISSENSE
FRAMESHIFT
PROTEININS
NONSENSE
PROTEINDEL

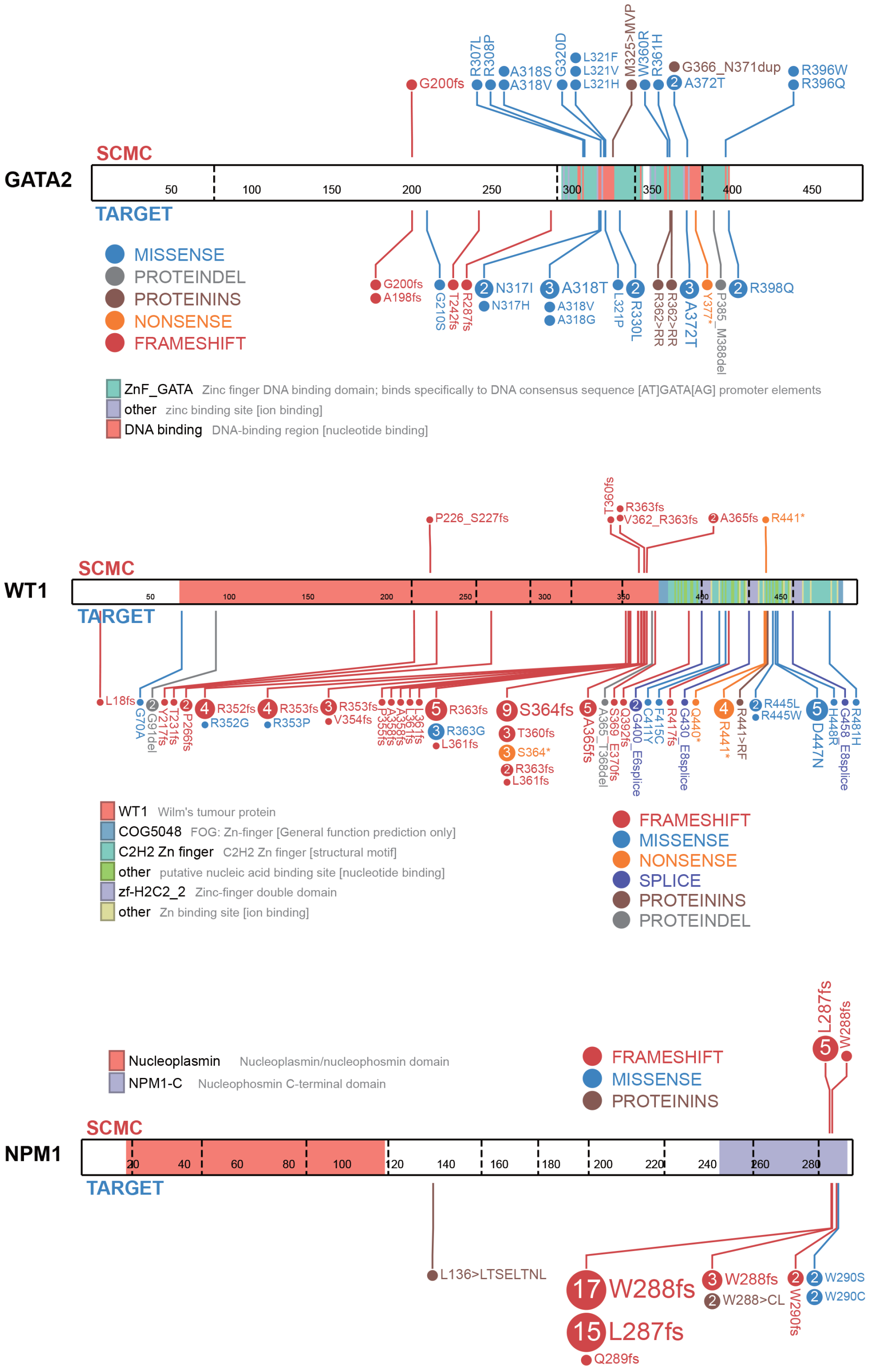
GATA2
SCMC
TARGET
G200fs
R307L
R308P
A318S
A318V
G320D
L321F
L321V
L321H
M325>MVP
W360R
R361H
G366_N371dup
A372T
R396W
R396Q
G200fs
A198fs
G210S
T242fs
R287fs
N317I
N317H
A318T
A318V
A318G
L321P
R330L
R362>RR
R362>RR
A372T
Y377*
P385_M388del
R398Q
MISSENSE
PROTEINDEL
PROTEININS
NONSENSE
FRAMESHIFT
ZnF_GATA Zinc finger DNA binding domain; binds specifically to DNA consensus sequence [AT]GATA[AG] promoter elements
other zinc binding site [ion binding]
DNA binding DNA-binding region [nucleotide binding]
WT1
SCMC
TARGET
P226_S227fs
T360fs
R363fs
V362_R363fs
A365fs
R441*
L18fs
G70A
G91del
R352fs
R352G
R353fs
R353P
R353fs
V354fs
R363fs
R363G
L361fs
S364fs
T360fs
S364*
R363fs
L361fs
A365fs
R441*
R441>RF
R445L
R445W
D447N
H448R
G458_E8splice
R481H
WT1 Wilm's tumour protein
COG5048 FOG: Zn-finger [General function prediction only]
C2H2 Zn finger C2H2 Zn finger [structural motif]
other putative nucleic acid binding site [nucleotide binding]
zf-H2C2_2 Zinc-finger double domain
other Zn binding site [ion binding]
FRAMESHIFT
MISSENSE
NONSENSE
SPLICE
PROTEININS
PROTEINDEL
Nucleoplasmin Nucleoplasmin/nucleophosmin domain
NPM1-C Nucleophosmin C-terminal domain
FRAMESHIFT
MISSENSE
PROTEININS
L287fs
W288fs
NPM1
SCMC
TARGET
L136>LTSELTNL
W288fs
L287fs
Q289fs
W288fs
W288>CL
W290fs
W290S
W290C

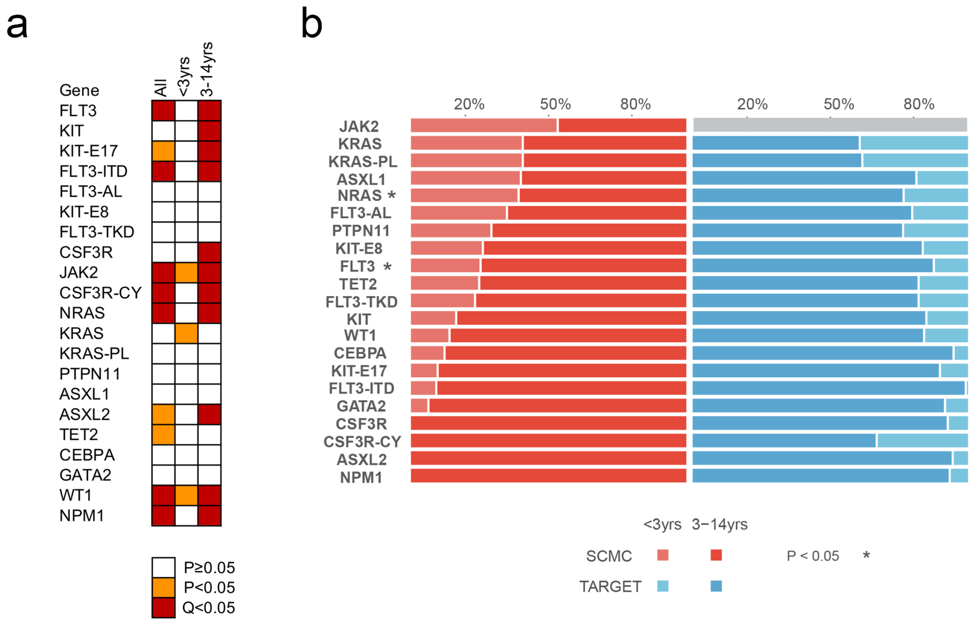


**Supplementary Figure 4. Comparison of mutation frequency by age group in Chinese (SCMC) and Western (TARGET) pediatric AML.** Genes or hotspots within driver gene with mutation frequency greater than 4% in either SCMC or TARGET cohort were included. Patients were breakdown into age groups of <3yrs and 3-14yrs. Mutation frequency between the two cohorts within each age group was compared (a). The composition of patients from two age groups were compared between SCMC and TARGET cohort and showed in (b). Two-sided Fisher's exact test was applied in this analysis.

a

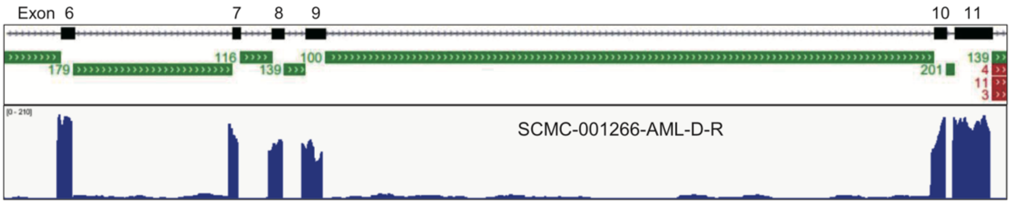


b

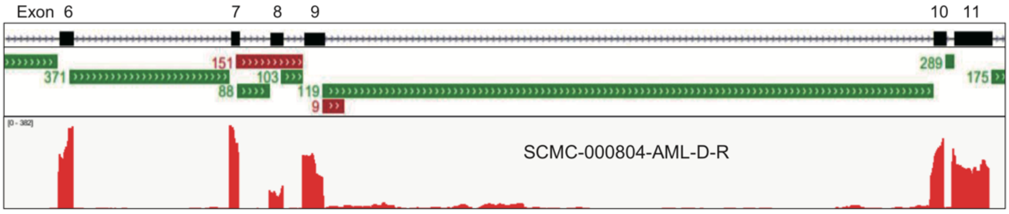


**Supplementary Figure 5. *CBL* exon8/9 deletions detected by RNA-seq in pediatric AML patients.** Junction analysis of *CBL* gene was applied and combined with coverage analysis to identify *CBL* exon8/9 deletion. Two cases were shown with IGV, as examples for *CBL* wild type (SCMC-001266-AML-D-R) in (**a**) and deletion (SCMC-000804-AML-D-R) in (**b**). In each panel, exon 6 to 11 of *CBL* gene was shown on the top, with the junctions between *CBL* exons showed in the middle and coverage plot from RNA-seq at the bottom. The wild type junctions were shown in green and abnormal ones in red. The number of reads representing each junction was labeled on left of each junction.

a

| | | SCMC | | | TARGET | | | P-value |
|---|---|---|---|---|---|---|---|---|
| | | Patients | 5-year EFS | 95%CI | Patients | 5-year EFS | 95%CI | (SCMC vs TARGET) |
| | All patients | n=288 | 49.20% | 0.435-0.557 | n=498 | 49.20% | 0.45-0.539 | 0.717 |
| Fusion | KMT2A rearrangements | n=43 | 56.30% | 0.428-0.74 | n=97 | 39.90% | 0.312-0.51 | 0.111 |
| | RUNX1-RUNX1T1 | n=82 | 55.30% | 0.448-0.682 | n=79 | 69.10% | 0.595-0.802 | 0.102 |
| | CBFA2T3-GLIS2 | n=12 | 16.70% | 0.047-0.591 | n=4 | NA | NA | 0.991 |
| | CBFB-MYH11 | n=10 | 80.00% | 0.587-1 | n=72 | 60.80% | 0.505-0.733 | 0.381 |
| | NUP98-NSD1 | n=5 | NA | NA | n=21 | 28.60% | 0.145-0.562 | 0.591 |
| | KMT2A-MLLT3 | n=16 | 64.80% | 0.436-0.962 | n=34 | 58.30% | 0.437-0.777 | 0.565 |
| | KMT2A-MLLT10 | n=10 | 40.00% | 0.187-0.855 | n=24 | 25.00% | 0.125-0.5 | 0.663 |
| Mutation | FLT3 | n=55 | 39.30% | 0.279-0.553 | n=158 | 45.90% | 0.386-0.545 | 0.288 |
| | KIT | n=51 | 41.70% | 0.29-0.598 | n=65 | 56.10% | 0.451-0.698 | 0.216 |
| | NRAS | n=50 | 42.50% | 0.305-0.592 | n=157 | 52.50% | 0.452-0.61 | 0.102 |
| | KRAS | n=27 | 58.80% | 0.428-0.809 | n=71 | 40.80% | 0.307-0.542 | 0.230 |
| | CEBPA | n=23 | 77.10% | 0.612-0.972 | n=36 | 59.50% | 0.452-0.784 | 0.211 |
| | PTPN11 | n=17 | 29.40% | 0.141-0.614 | n=42 | 44.20% | 0.314-0.624 | 0.271 |

b

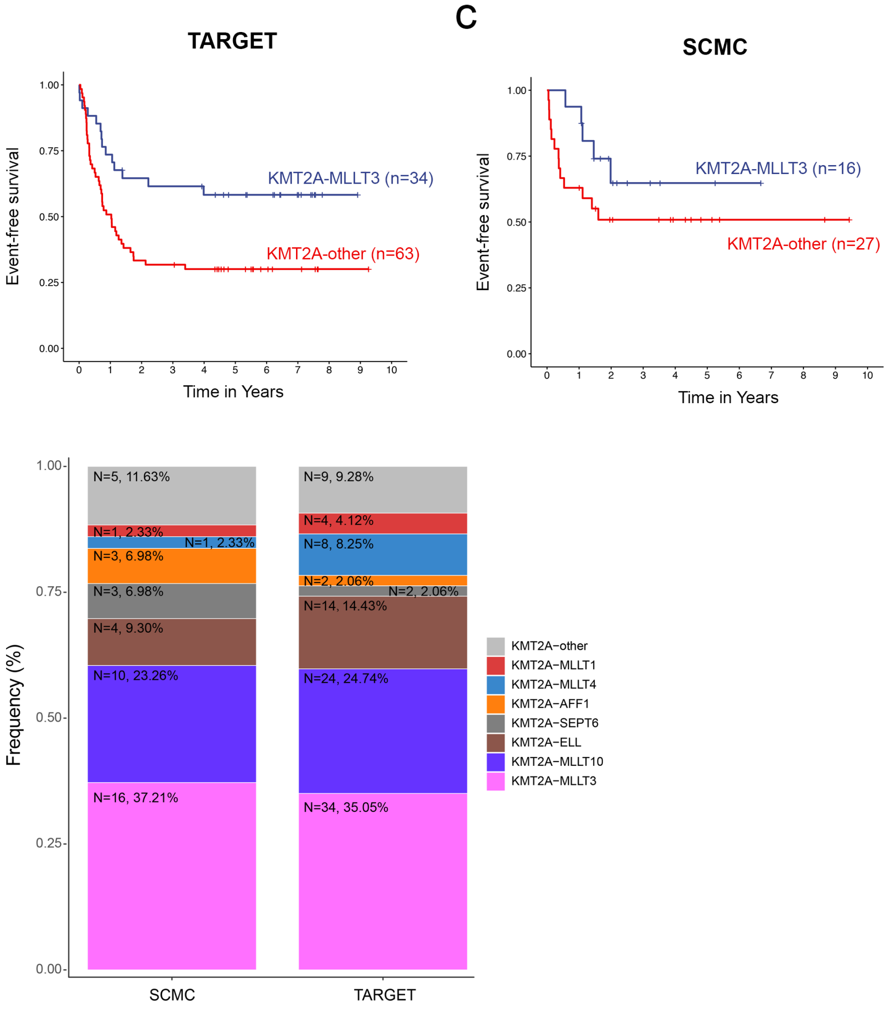


**Supplementary Figure 6. Clinical impact of driver genomic aberrations in Chinese (SCMC) and Western (TARGET) pediatric AML.** Only patients younger than 15-year-old were included. (**a**) Event-free survival analysis of cases carrying driver fusions and mutations between SCMC and TARGET cohort. Patients without sufficient follow-up information was excluded from this analysis (n=3 for *KIT*, n=1 for *NRAS* and *CEBPA* each). (**b**) and (**c**) Event-free survival analysis of AMLs carrying

*KMT2A-MLLT3* fusion versus other *KMT2A* rearrangements in TARGET and SCMC cohort, respectively. (**d**) The constitution of *KMT2A* rearrangements in SCMC (left) and TARGET (right). *P* values were calculated with log-rank test.

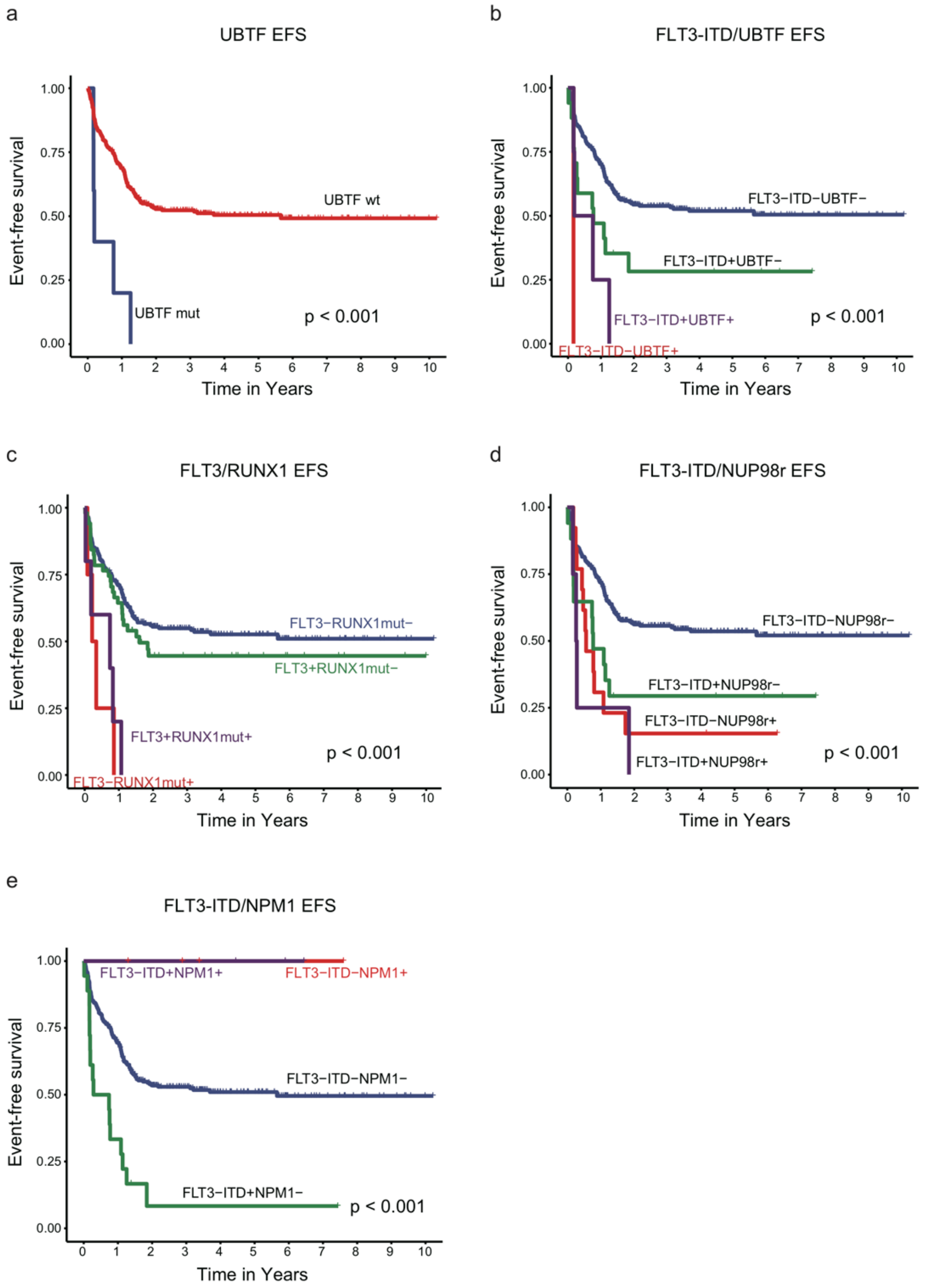


**Supplementary Figure 7. Clinical impact of genetic alterations on event free survival of AML patients.** (**a**) Kaplan-Meier estimates of EFS of Chinese AML patients with *UBTF* mutation (n = 5) and wild type (n = 283). *P* = 3.712E-04. (**b**) Kaplan-Meier estimates of EFS of patients carrying both *FLT3*-ITD and *UBTF* mutation (n = 4); *FLT3*-ITD alone (n = 17); *UBTF* alone (n = 1) and wild type for both (n = 266). *P* = 6.737E−05 (**c**) Kaplan-Meier estimates of EFS of patients with both *FLT3* (including ITD) and *RUNX1* sequence mutation (n = 5); *FLT3* mutation alone (n = 51); *RUNX1* alone (n = 4) and wild type for both (n = 228). *P* = 3.553E−06. (**d**) Kaplan-Meier estimates of EFS

of patients with *FLT3*-ITD and *NUP98* rearrangement (n = 4); *FLT3*-ITD alone (n = 17); *NUP98* rearrangement alone (n = 13) and wild type for both (n = 254). *P* = 2.561E−05. (**e**) Kaplan-Meier estimates of EFS of patients with *FLT3*-ITD and *NPM1* mutation (n = 3); *FLT3*-ITD alone (n = 18); *NPM1* mutation alone (n = 4) and wild type for both (n = 263). *P* = 4.609E−06. *P* values were calculated with log-rank test in each panel.

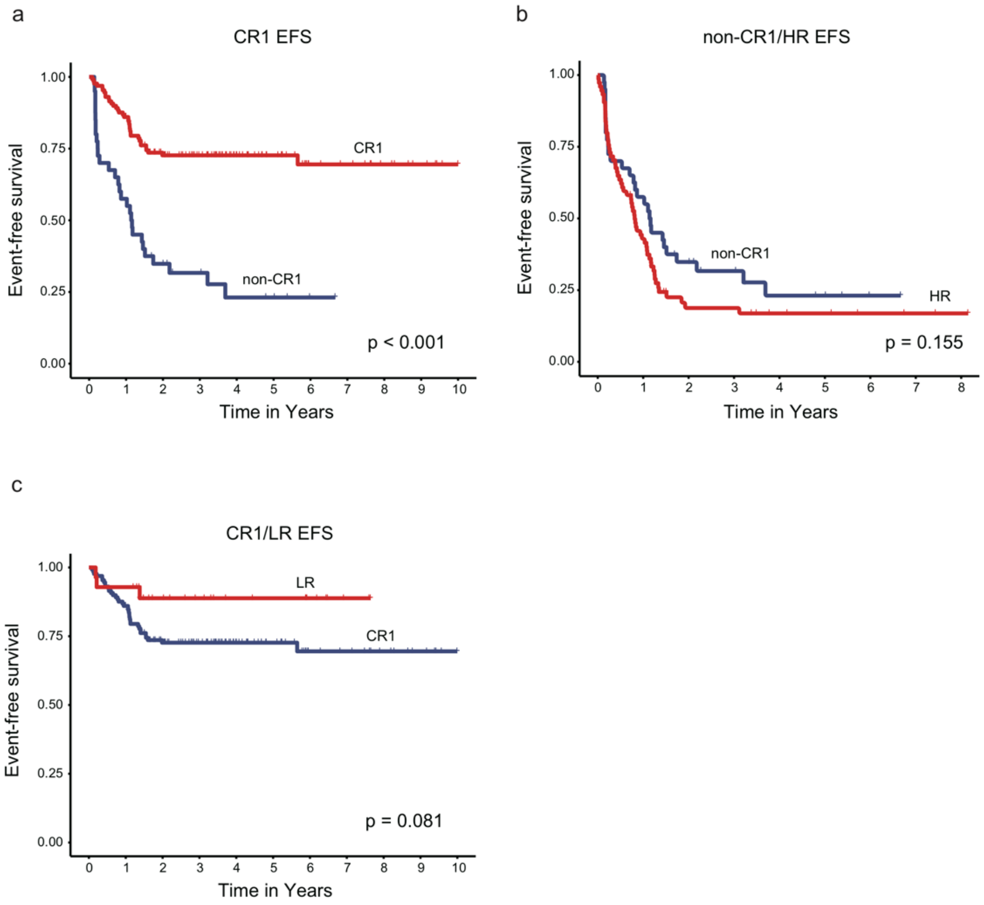


**Supplementary Figure 8. Impact of treatment response on event free survival of patients negative for prognosis associated genomic alteration.** (**a**) Kaplan-Meier estimates of EFS of Chinese AML patients reached complete response in 1st round treatment (CR1, n = 129) or not (non-CR1, n = 40). *P* = 1.283E−08. (**b**) Kaplan-Meier estimates of EFS of non-CR1 patients (n = 40) and high risk patients (HR, n = 74) defined as positive for genomic alterations associated with inferior outcome (Results and Supplementary Table 12). (**c**) Kaplan-Meier estimates of EFS of CR1 patients (n = 129) and low risk patients (LR, n = 28) defined as carrying genetic alterations associated with favorable outcome (Results and Supplementary Table 12). *P* values were calculated with log-rank test in each panel.

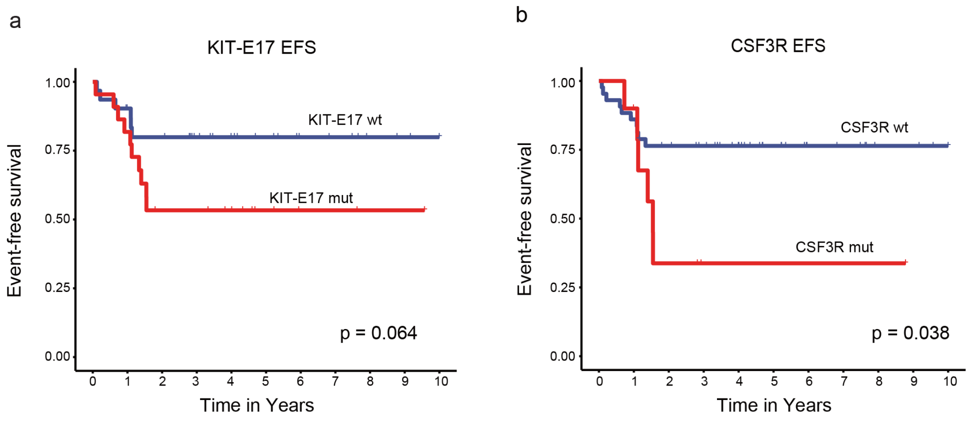


**Supplementary Figure 9. Impact of *KIT*-E17 and *CSF3R* mutations on event free survival of CR1 *RUNX1-RUNX1T1* positive AML patients.** (**a**) Kaplan-Meier estimates of EFS of CR1 *RUNX1-RUNX1T1* positive AML patients with *KIT*-E17 mutation (n = 22) or wild type (n = 31). (**b**) Kaplan-Meier estimates of CR1 patients carrying *CSF3R* mutation (n = 10) or not (n = 43). *P* values were calculated with log-rank test in each panel.

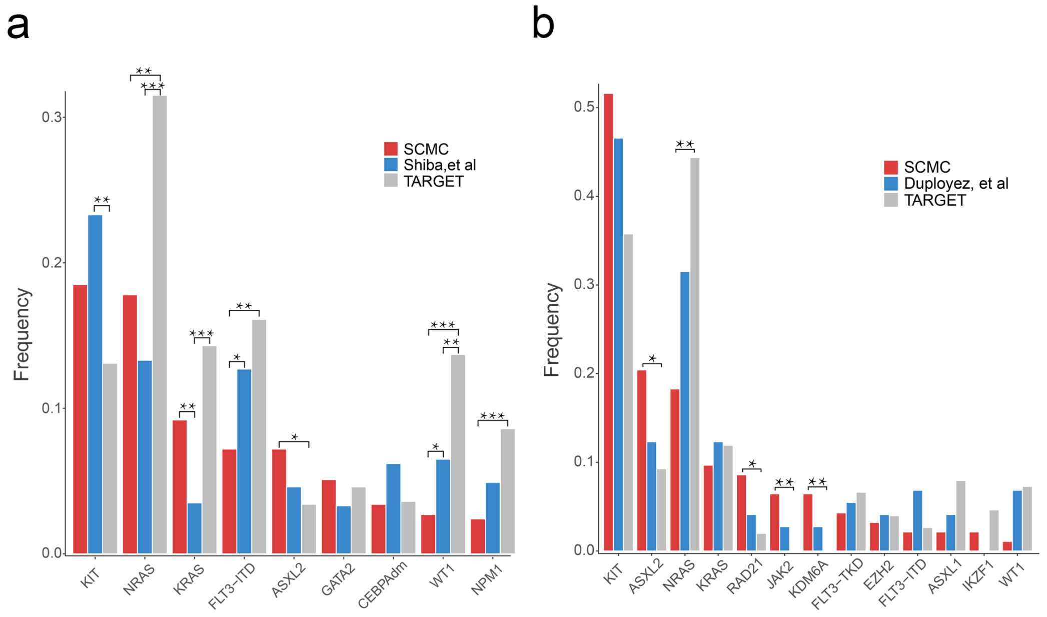


**Supplementary Figure 10. Comparison of driver mutation recurrence between East and West pediatric AML cohorts.** Genomic aberrations reported by Shiba et al and Duployez et al were analyzed, representing Japanese pediatric AML (**a**) and French pediatric CBF AML cohort (**b**) respectively. Patients under 18-year-old were included in this analysis. * $P < 0.05$, ** $P < 0.01$, *** $P < 0.001$, two-sided Fisher's exact test. Exact $P$ values for each comparison were present in Supplementary Data 16.

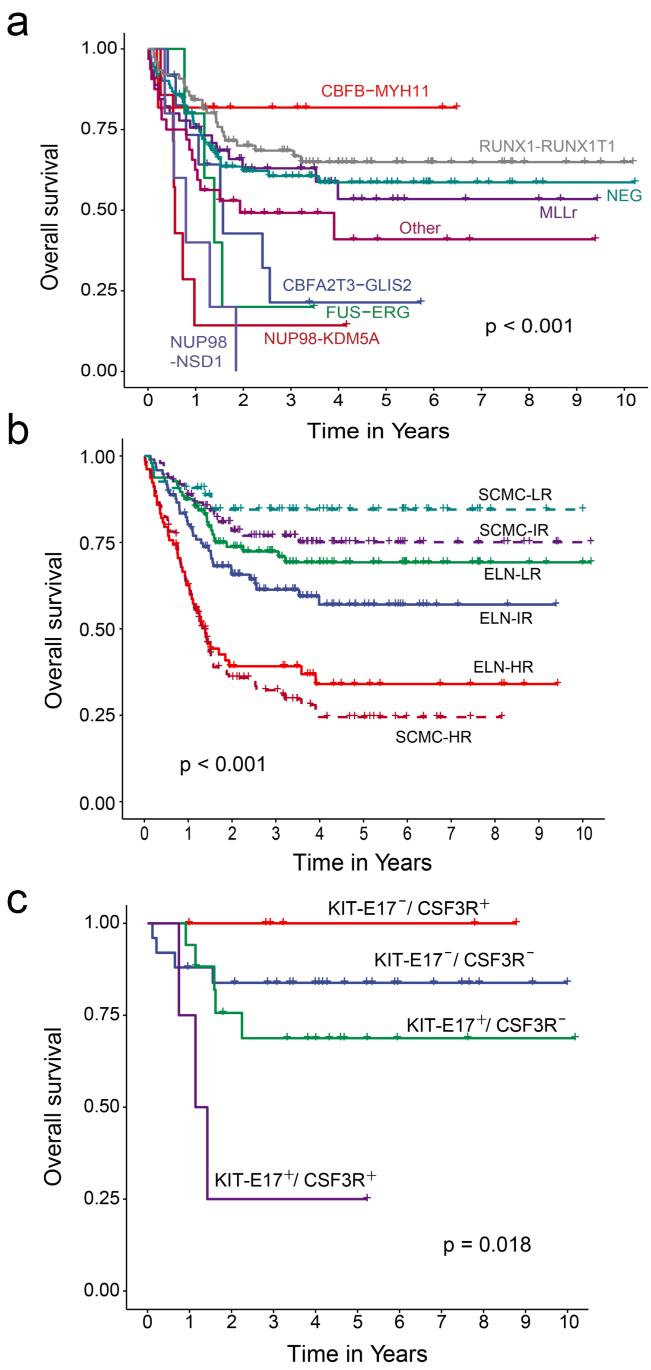


**Supplementary Figure 11. Overall survival (OS) analysis of Chinese pediatric AML.** Kaplan-Meier estimate the OS of Chinese pediatric AML patients diagnosed and treated at SCMC, grouped by fusions (**a**) (*P* = 4.700E−05); risk groups as stratified by revised SCMC-pAML or ELN model (**b**) (*P* = 8.095E−20); and *CSF3R* and *KIT*-E17 mutation status within *RUNX1-RUNX1T1* positive patients reached complete remission after the 1st cycle of induction therapy (**c**). *P* values were calculated with log-rank test in each panel.
