# Supplementary material for: Distinct genomic landscape of Chinese pediatric acute myeloid leukemia impacts clinical risk classification
Source: Nat Commun. 2022 Mar 28;13:1640. doi: 10.1038/s41467-022-29336-y (PMC8960760; doi:10.1038/s41467-022-29336-y)
Supplement: Supplementary file 2 — Description of Additional Supplementary Files [file 41467_2022_29336_MOESM2_ESM.pdf]

Description of Additional Supplementary Files

File Name: Supplementary Data 1
Description: Patient Information.

File Name: Supplementary Data 2
Description: Clinical characterization of SCMC and TARGET AML cohort.

File Name: Supplementary Data 3
Description: QC of RNAseq data.

File Name: Supplementary Data 4
Description: Fusions identified from RNA-seq.

File Name: Supplementary Data 5
Description: Comparison of mutations discovered from WGS and RNA-seq.

File Name: Supplementary Data 6
Description: Sequence mutations identified from RNA-seq analysis.

File Name: Supplementary Data 7
Description: Internal Tandem Duplication identified from RNA-seq analysis.

File Name: Supplementary Data 8
Description:

a. Comparison of mutation frequencies in AML between East and West population.
b. Mutation composition in different age groups

File Name: Supplementary Data 9
Description: CBL deletions.

File Name: Supplementary Data 10
Description: Driver genes and the associated pathways.

File Name: Supplementary Data 11
Description: Pairwise association between driver genes.

File Name: Supplementary Data 12
Description: Associations between driver mutations and eventfree survival.

File Name: Supplementary Data 13
Description:

a. Univariate Cox analysis.
b. Multivariate Cox analysis.

File Name: Supplementary Data 14
Description: SCMC-pAML model for risk stratification.

File Name: Supplementary Data 15
Description: Multivariable Cox models for different risk group classification.

File Name: Supplementary Data 16
Description: Comparison of genomic aberrations among different pediatric AML cohort.
