# Supplementary material for: Distinct genomic landscape of Chinese pediatric acute myeloid leukemia impacts clinical risk classification
Source: Nat Commun. 2022 Mar 28;13:1640. doi: 10.1038/s41467-022-29336-y (PMC8960760; doi:10.1038/s41467-022-29336-y)
Supplement: Supplementary file 21 — Reporting Summary [file 41467_2022_29336_MOESM21_ESM.pdf]

nature portfolio

Corresponding author(s): Yu Liu and Shuhong Shen

Last updated by author(s): Mar 8, 2022

Reporting Summary

Nature Portfolio wishes to improve the reproducibility of the work that we publish. This form provides structure for consistency and transparency in reporting. For further information on Nature Portfolio policies, see our Editorial Policies and the Editorial Policy Checklist.

Statistics

For all statistical analyses, confirm that the following items are present in the figure legend, table legend, main text, or Methods section.

n/a	Confirmed	
☐	☒	The exact sample size (*n*) for each experimental group/condition, given as a discrete number and unit of measurement
☒	☐	A statement on whether measurements were taken from distinct samples or whether the same sample was measured repeatedly
☐	☒	The statistical test(s) used AND whether they are one- or two-sided<br>*Only common tests should be described solely by name; describe more complex techniques in the Methods section.*
☒	☐	A description of all covariates tested
☐	☒	A description of any assumptions or corrections, such as tests of normality and adjustment for multiple comparisons
☐	☒	A full description of the statistical parameters including central tendency (e.g. means) or other basic estimates (e.g. regression coefficient) AND variation (e.g. standard deviation) or associated estimates of uncertainty (e.g. confidence intervals)
☐	☒	For null hypothesis testing, the test statistic (e.g. *F*, *t*, *r*) with confidence intervals, effect sizes, degrees of freedom and *P* value noted<br>*Give P values as exact values whenever suitable.*
☒	☐	For Bayesian analysis, information on the choice of priors and Markov chain Monte Carlo settings
☒	☐	For hierarchical and complex designs, identification of the appropriate level for tests and full reporting of outcomes
☒	☐	Estimates of effect sizes (e.g. Cohen's *d*, Pearson's *r*), indicating how they were calculated

Our web collection on statistics for biologists contains articles on many of the points above.

Software and code

Policy information about availability of computer code

Data collection	No software was used. The somatic mutations of pediatric tumors used in MutClan were collected from St. Jude Cloud (https://www.stjude.cloud/). Mutations from WGS of 10 previously published pediatric ALL were collected from supplementary table of the published manuscript (PMID: 31697823). The multi-platform verified somatic variants from TARGET dataset was obtained from Bolouri et al (PMID: 29227476). The genomic mutations in Japanese pediatric AML cohort was obtained from Shiba et al (PMID: 31648321). Mutations from CBF-AML representing French cohort was collected from Duployez et al (PMID: 26980726).
Data analysis	RNA-seq data was mapped with STAR (2.7.1a) and marked duplicate with picard (2.22.9). Fusion was analyzed with CICERO (v.0.16) and FusionCatcher (v1.00). Mutation was analyzed with MuTect2 and RNAIndel (2.0.0) and annotated with VEP (v97.3). Driver mutation analysis was done with PeCanPIE (https://pecan.stjude.cloud/pie) and MutClan (Manuscript in preparation). In house python code was used in filtering and processing the sequence mutations. Statistical analyses were performed with Rstudio (v4.1.0).

For manuscripts utilizing custom algorithms or software that are central to the research but not yet described in published literature, software must be made available to editors and reviewers. We strongly encourage code deposition in a community repository (e.g. GitHub). See the Nature Portfolio guidelines for submitting code & software for further information.

Data

Policy information about availability of data

All manuscripts must include a data availability statement. This statement should provide the following information, where applicable:

- Accession codes, unique identifiers, or web links for publicly available datasets
- A description of any restrictions on data availability
- For clinical datasets or third party data, please ensure that the statement adheres to our policy

The RNA-seq data generated in this study have been deposited in the Genome Sequence Archive (GSA) for Human of the National Genomics Data Center of China under accession number HRA000789 (https://bigd.big.ac.cn/gsa-human/browse/HRA000789). The data is available for academic use under controlled access in compliance with the regulation of the Ministry of Science and Technology (MOST) of China for the deposit and use of human genomic data. Access can be obtained by contacting members of the Data Access Committee (DAC) Shuhong Shen at shenshuhong@scmc.com.cn or Yu Liu at liuyu@scmc.com.cn and following the application procedure in GSA. For detailed guidance, see GSA-Human_Request_Guide_for_Users [https://ngdc.cncb.ac.cn/gsa-human/document/GSA-Human_Request_Guide_for_Users_us.pdf]. Data will be available immediately once the application was approved. The access to the controlled data will be valid for one year from the date approved. The processed genomic aberrations from this dataset are available within the Supplementary Information files. The publicly available genomic data for TARGET AML are available in the database of Genotypes and Phenotypes (dbGap) under accession number phs000465 (https://www.ncbi.nlm.nih.gov/projects/gap/cgi-bin/study.cgi?study_id=phs000465.v21.p8). The clinical annotations of TARGET AML cohort and verified somatic genomic variants from both discovery and validation dataset are downloaded from Bolouri et al, (PMID: 29227476) and TARGET Data Matrix (https://ocg.cancer.gov/programs/target/data-matrix). The 10 previously published RNA-seq data re-analyzed in this study are available as part in GSA for Human under accession number HRA000119 (https://bigd.big.ac.cn/gsa-human/browse/HRA000119). The mutations from Japanese and French pediatric AML cohorts were obtained from Shiba et al (PMID: 31648321) and Duployez et al (PMID: 26980726) respectively. The remaining data are available within the Article or Supplementary Information.

Field-specific reporting

Please select the one below that is the best fit for your research. If you are not sure, read the appropriate sections before making your selection.

☒ Life sciences ☐ Behavioural & social sciences ☐ Ecological, evolutionary & environmental sciences

For a reference copy of the document with all sections, see nature.com/documents/nr-reporting-summary-flat.pdf

Life sciences study design

All studies must disclose on these points even when the disclosure is negative.

Sample size	We analyzed all pediatric AML cases diagnosed and treated at Shanghai Children's Medical Center (SCMC) from 2001–2018, with adequate material from tumor cells was available.
Data exclusions	Four cases were excluded from the survival analysis due to insufficient follow up information. Twenty-one cases were excluded from risk category re-stratifying due to missing information regarding treatment response of first round of induction treatment. The number of cases included in each analysis was described accordingly.
Replication	The findings of different mutation profile between East and West pediatric AML were replicated with independent AML cohort (Shiba et al, PMID: 31648321; Duployez et al, PMID: 26980726).
Randomization	We didn't randomly select participants in this study. We applied RNA-seq experiment and following analysis to all the cases diagnosed as AML and treated at SCMC from 2001-2018, with adequate material available.
Blinding	We analyzed all cases diagnosed as AML and treated at SCMC from 2001-2018, with adequate material available. The RNA-seq experiments and genomic analysis was performed blinded to all clinical information.

Reporting for specific materials, systems and methods

We require information from authors about some types of materials, experimental systems and methods used in many studies. Here, indicate whether each material, system or method listed is relevant to your study. If you are not sure if a list item applies to your research, read the appropriate section before selecting a response.

Materials & experimental systems

n/a	Involved in the study
☒	☐ Antibodies
☒	☐ Eukaryotic cell lines
☒	☐ Palaeontology and archaeology
☒	☐ Animals and other organisms
☐	☒ Human research participants
☒	☐ Clinical data
☒	☐ Dual use research of concern

Methods

n/a	Involved in the study
☒	☐ ChIP-seq
☒	☐ Flow cytometry
☒	☐ MRI-based neuroimaging

Human research participants

Policy information about studies involving human research participants

Population characteristics	A total of 292 pediatric AML patients were included in this study, including 117 female and 175 male; 107 patients was diagnosed under age of 3, age 3-10 years: 111, 10-15 years: 70 and 15-18 years: 4.
Recruitment	We included all patients diagnosed as AML and treated at SCMC from 2001-2018 in this study and performed RNA-seq for the cases with adequate material available. No future selection was applied in this study.
Ethics oversight	The research was approved by the Ethics Committee at Shanghai Children's Medical Center (SCMC).

Note that full information on the approval of the study protocol must also be provided in the manuscript.
